# Search and Match Task: Development of a Taskified Match-3 Puzzle Game to Assess and Practice Visual Search

**DOI:** 10.2196/13620

**Published:** 2019-05-09

**Authors:** Alvin Chesham, Stephan Moreno Gerber, Narayan Schütz, Hugo Saner, Klemens Gutbrod, René Martin Müri, Tobias Nef, Prabitha Urwyler

**Affiliations:** 1 Gerontechnology & Rehabilitation University of Bern Bern Switzerland; 2 Department of Cardiology University Hospital (Inselspital) Bern Switzerland; 3 Department of Neurology, University Neurorehabilitation University Hospital Bern (Inselspital) University of Bern Bern Switzerland; 4 Artificial Organ Center for Biomedical Engineering Research University of Bern Bern Switzerland

**Keywords:** match-three puzzle games, video games, task difficulty, attention, pattern recognition, visual, aging, neuropsychological tests

## Abstract

**Background:**

Visual search declines with aging, dementia, and brain injury and is linked to limitations in everyday activities. Recent studies suggest that visual search can be improved with practice using computerized visual search tasks and puzzle video games. For practical use, it is important that visual search ability can be assessed and practiced in a controlled and adaptive way. However, commercial puzzle video games make it hard to control task difficulty, and there are little means to collect performance data.

**Objective:**

The aim of this study was to develop and initially validate the search and match task (SMT) that combines an enjoyable tile-matching match-3 puzzle video game with features of the visual search paradigm (*taskified game*). The SMT was designed as a single-target visual search task that allows control over task difficulty variables and collection of performance data.

**Methods:**

The SMT is played on a grid-based (width × height) puzzle board, filled with different types of colored polygons. A wide range of difficulty levels was generated by combinations of 3 task variables over a range from 4 to 8 including height and width of the puzzle board (set size) and the numbers of tile types (distractor heterogeneity). For each difficulty level, large numbers of playable trials were pregenerated using Python. Each trial consists of 4 consecutive puzzle boards, where the goal of the task is to find a target tile configuration (*search*) on the puzzle board and swap 2 adjacent tiles to create a line of 3 identical tiles (*match*). For each puzzle board, there is exactly 1 possible match (*single target* search). In a user study with 28 young adults (aged 18 to 31 years), 13 older (aged 64 to 79 years) and 11 oldest (aged 86 to 98 years) adults played the long (young and older adults) or short version (oldest adults) of the difficulty levels of the SMT. Participants rated their perception and the usability of the task and completed neuropsychological tests that measure cognitive domains engaged by the puzzle game.

**Results:**

Results from the user study indicate that the target search time is associated with set size, distractor heterogeneity, and age. Results further indicate that search performance is associated with general cognitive ability, selective and divided attention, visual search, and visuospatial and pattern recognition ability.

**Conclusions:**

Overall, this study shows that an everyday puzzle game–based task can be experimentally controlled, is enjoyable and user-friendly, and permits data collection to assess visual search and cognitive abilities. Further research is needed to evaluate the potential of the SMT game to assess and practice visual search ability in an enjoyable and adaptive way. A PsychoPy version of the SMT is freely available for researchers.

## Introduction

Visual search is the ability to find target objects in complex visual scenes in everyday life [1]. Search skills are usually assessed with visual search tasks, where a target stimulus is presented among distractor stimuli on a display. The number of stimuli on the display (set size) and perceptual dimension of the stimuli are varied to manipulate the complexity of visual search tasks [2]. More complex visual search is often affected in aging, in neurodegenerative diseases, and after brain injury [3]. Studies indicate that visual search can be improved following training on visual search tasks [4] and match-3 puzzle video games [5,6]. Tile-matching match-3 (TMM3) puzzle video games require finding and matching 3 tiles of the same type on a board of tiles that differ on some dimensions. The aim of this study was to develop and initially validate a TMM3 puzzle video game that engages visual search ability in a playful and engaging way, permits control over task difficulty parameters, and enables collection of data useful for researchers.

### Traditional Visual Search Tasks

Visual search is required to detect a behaviorally relevant object among a set of irrelevant objects by scanning the visual environment that is important in both everyday activities (eg, finding an item on a supermarket shelf) and professional settings (eg, searching medical images for signs of abnormalities) [1,7]. Visual search is usually assessed with experimentally controlled visual search tasks that represent a suitable measure of everyday search ability [8]. In the visual search paradigm, participants are asked to detect a target stimulus defined by basic visual features (eg, color and shape) and whose presence and location are unknown, among a set of distractors (nontarget) as quickly and accurately as possible [9-11]. Overall, 2 independent variables are used to manipulate search difficulty: the total number of items on the display (set size) and the perceptual dimensions (eg, color and shape) affecting the similarity between target and distractors (target-distractor similarity) and among distractors (distractor heterogeneity) [2]. As a dependent variable, 2 measures of search performance are calculated: search time and search efficiency. Search time is measured by overall reaction time (RT), whereas search efficiency is calculated as processing time per search item. Search efficiency is derived from the slope of RTs as a function of set size (RT × set size) [2,12].

### Variations and Types of Visual Search Tasks

**Figure 1:**
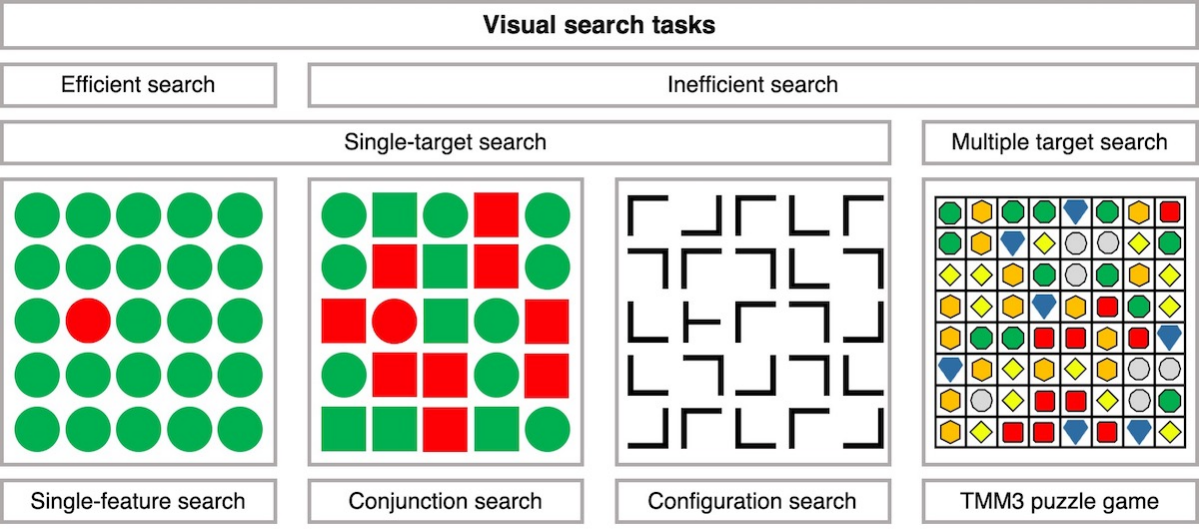
Types of visual search tasks and tile-matching match-three puzzle games. Efficient feature search where a single target (red circle) is shown among distractors (green circles) that differ in a single feature (color). Inefficient conjunction search where a single target (red circle) is presented among distractors (red squares, green circles and green squares) that share one of two target features (color or shape). In efficient configuration search (T among L) where a single target (T) is hidden among distractors (L in 4 orientations), that share the same basic features (black vertical and horizontal lines) but differ in their configuration. Controlled tile-matching match-3 puzzle game where multiple spatial configurations of three or more identical tiles must be found. These target configurations can be turned in to a line of three by swapping 2 adjacent tiles.

Visual search tasks vary in search efficiency depending on the number of perceptual dimensions that affect target-distractor similarity and distractor heterogeneity [2,13]. In efficient search tasks, the target differs from distractor items by a single basic feature (Figure 1, *Single-feature Search*). Efficient search is driven by perceptual bottom-up processes and independent of the number of items in the search display (set size). As feature search depends on the similarity between the target and distractors, single-feature search becomes less efficient with increasing target-distractor similarity and distractor heterogeneity [13]. However, in everyday life, search items often consist of specific conjunctions or spatial configurations of visual features that are more difficult to detect than single features [10]. Inefficient search tasks include conjunction and configuration search. In conjunction search tasks, targets are defined by a combination of 2 features among distracting items that share only one of these 2 features (Figure 1, *Conjunction Search*). In spatial configuration search tasks, the target consists of a specific spatial arrangement of features. Although targets and distractors are composed of the same elements, they differ in their spatial configuration (Figure 1, *Configuration Search*) [14]. In inefficient search, targets differ from distractors in more than one feature dimension and need to be attended item by item. This requires attentional top-down control such that increased set size leads to prolonged search [2,15].

### Visual Search in Aging, Neurodegenerative Diseases, and Brain Injury

More effortful visual search can become increasingly challenging with normal aging, neurodegenerative diseases, including Alzheimer's disease (AD) and Parkinson’s disease, and after brain injury [3,16-18]. A general finding is that there is an exaggerated cost of increased set size on search time in search tasks where more than one perceptual dimension defines the target (inefficient search). Deficits in inefficient visual search were shown to deteriorate progressively from young to older adults [19-21], to mild cognitive impairment (MCI) [17,22,23], to MCI-AD converters compared with non-AD converters [24], and to patients with AD [17,23,25,26]. These findings indicate the role of visual search tasks as an indicator of age-related neuropathological changes in brain areas supporting visual search that are not usually assessed in clinical practice. Visual search is supported by frontoparietal attentional networks that are particularly vulnerable to neurodegenerative disorders [17,21]. Damage to these brain areas has also been linked with visual search deficits after traumatic brain injury [27] and stroke [28]. Although not routinely assessed in clinical practice, deficits in inefficient visual search are linked with long-term limitations in everyday activities that involve visual search [29].

### Visual Search Training

Owing to the predominant role of visual search in everyday life, it is important to assess and practice visual search abilities [28]. Throughout their lifetime, humans learn combinations of visual object features (ie, conjunction and spatial relations) to optimize searching for specific objects in their everyday visual environment [30,31]. Studies have shown that younger and older adults can increase visual search ability through repeated practice on conjunction and configuration search tasks [32-34]. There is controversy whether training effects reflect low-level learning (*feature learning*) that is specific to the trained task and stimuli or high-level learning (*conjunction learning*) that is more general and transferable [35]. However, visual search training benefits were shown to be generalizable and proposed to combine both types of learning that make it important for improving visual search in everyday life [30,31,36]. On the basis of these findings, 2 new approaches to improving visual search have been taken. In the first, studies have used theory-driven computerized conjunction search tasks to both assess and improve visual search abilities. The advantage of computerized visual search tasks is that assessments and trainings can be flexibly adjusted by manipulating parameters of the task based on performance measures. Task difficulty is mainly accomplished by manipulating 2 task parameters: the total number of stimuli on the screen (set size) and the variation or heterogeneity in distractor stimuli that affect target-distractor and distractor-distractor similarity [37-39].

In a second approach, TMM3 puzzle video games (see Figure 1, *TMM3 Puzzle Game*) have been used to practice visual search ability. Recent studies showed improvements in visual search in both healthy younger and older adults after training with a TMM3 puzzle game [5,6,40,41]. This shows that puzzle games that include a search element can be used to train visual search ability. The advantage of using puzzle games is that they are highly popular, easy to learn and play, and are particularly liked by older adults [42,43]. This underlines the potential of puzzle games as a nonthreatening and enjoyable way to assess and practice visual search and cognitive function in older adults [44]. However, commercial games make it hard to control variables that affect the task difficulty, and usually, there are little means to collect data for research purposes [45].

Overall, these findings show the potential of both computerized visual search tasks and puzzle video games as means to assess and practice visual search ability. To combine the strengths of these 2 approaches, games can be modified or rewritten as *game-like tasks* or *taskified games* that can be used as valid cognitive tests and interventions while keeping all the elements of a video game [45,46]. As visual search is not routinely assessed in clinical settings, new user-friendly tools that permit assessing and practicing visual search ability in a controlled and gradable fashion are clearly needed [28,29].

### Visual Search and Tile-Matching Match-3 Puzzle Games

**Figure 2:**
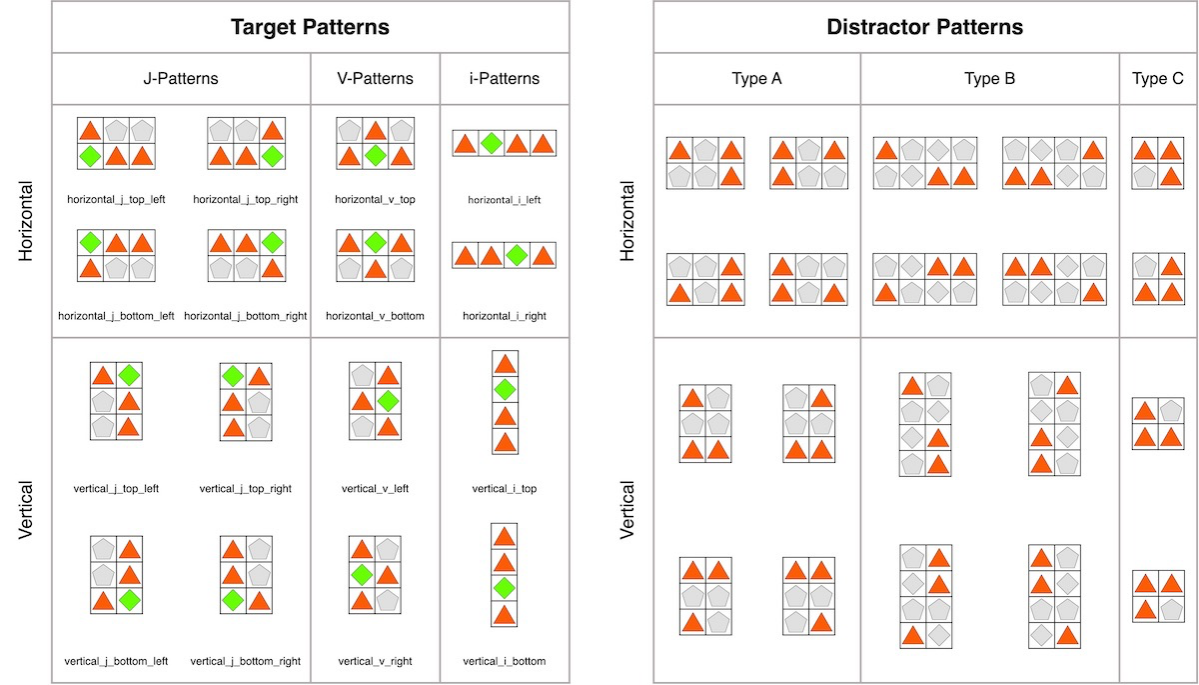
Target pattern categories (left): The green tile can be swapped with the respective opposite red tile to make a line of 3 red tiles (match). There are 3 basic target patterns that can be matched by moving a tile diagonal from a pair of identical pieces (J-patterns), between 2 identical tiles (V-patterns) and toward a pair of tiles (i-patterns). There are 16 different types of target patterns. Distractor pattern categories (right): Distractor patterns (red tiles) are false target patterns with 2 adjacent tiles and a third tile that deviates by 1 cell from the 3 basic target patterns. Type A and C patterns are distractors of J and V target patterns, whereas type B patterns are distractors of i and J target patterns. There are a total 20 possible distractor patterns.

The 2 constituent elements of TMM3 games are a puzzle board and colored shapes. The puzzle board is a rectangular or square grid, and each cell inside the grid contains a colored shape (tile). In classical TMM3 games, the goal is to eliminate as many tiles as possible in a limited time period [47]. To eliminate tiles, groups of 3 or more identical tiles must be found and aligned by exchanging the position of 2 adjacent tiles (*match*). The matched tiles are then removed, and new tiles fall in their place [48] (see Figure 1, *TMM3 puzzle game*, and Figure 2). The difficulty in TMM3 games is increased when the number of potential matches on the puzzle board decreases, making it harder to find tiles to eliminate [6].

TMM3 puzzle games combine visual search with visual pattern recognition and matching [49]. Search targets are defined by 2 features: the color and shape of the tiles and the spatial relation among them. Unlike visual search tasks, targets are always present because the goal of TMM3 games is to continuously make matches and eliminate tiles [5]. It should be noted that TMM3 puzzle games combine elements from inefficient visual search tasks (see Figure 1, *TMM3 Puzzle Game*). Similar to configuration search, the goal is to find a group or spatial arrangement of 3 identical tiles. As in conjunction search, the target is made up of a combination or conjunction of features: a visual feature (3 identical tiles) and spatial feature (target pattern configuration) [6].

**Figure 3:**
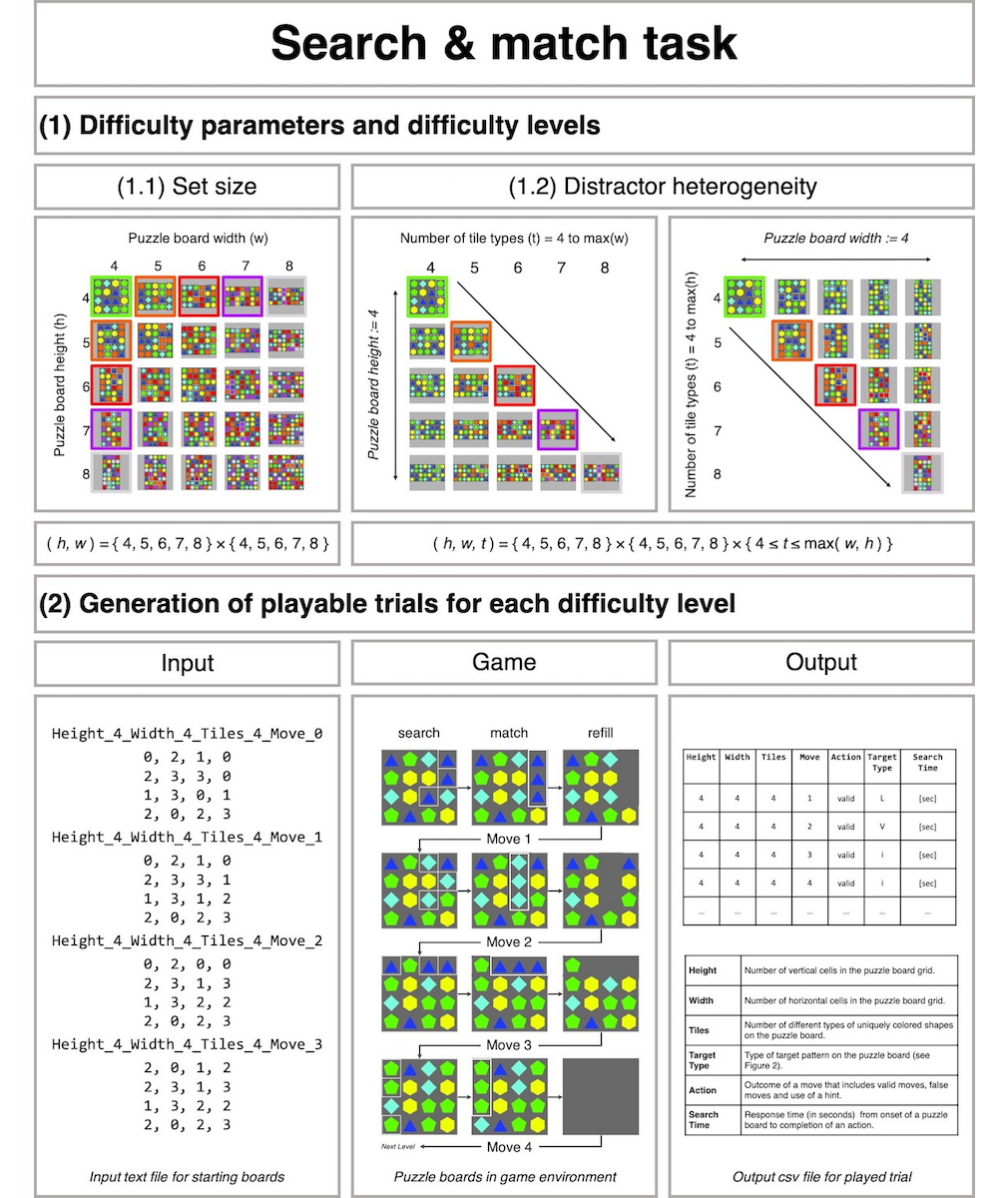
Difficulty parameters and development of the search & match task. (1) Difficulty parameters and difficulty levels include the set size, ie, height and width of the puzzle board, and distractor heterogeneity, ie, the number of different types of colored shapes. (1.1) Set size. Difficulty levels were first generated by creating combinations of puzzle board with widths and heights ranging from 4 to 8. (1.2) Distractor heterogeneity. For each set size, distractor heterogeneity was manipulated from 4 to the maximum value of either height or width. Examples are shown for width and height ranging from 4 to 8, with height and width set fixed at a value of 4. (2) Generation of playable trials for each difficulty level. For each difficulty level, playable trials were pregenerated as text files (Input). A trial consists of 4 consecutive puzzle boards with 1 single target pattern (Game - search). After swapping each target pattern (Game - match), the tiles are removed and replaced with new tiles (Game - refill). Trials were programmed such that after each refill there was only 1 single target pattern. Performance data was recorded at move level (Output). For further information on data collected in the Search & Match Task, see the Search & Match Task Instruction file.

In TMM3 games, there are both multiple possible targets on the puzzle board (multiple target search) and multiple types of targets (multiple category search; see Figure 2). In TMM3 puzzles, there are 16 target patterns based on 3 basic types of patterns (see Figure 2, left) [50,52–54]. Each target pattern is defined by a specific spatial relation among 3 identical tiles. Distractors in TMM3 puzzles are called false target or distractor patterns that are almost like target patterns but create no match (see Figure 3, right). Therefore, TMM3 puzzles require spatial attention and pattern recognition to discriminate targets from distractor patterns. This search is similar to inefficient visual search because spatial relations among tiles must be compared item by item until a target pattern is found [5,6,50]. A proposed search mechanism for TMM3 games is to first look for 2 adjacent tiles of the same color and shape (*find two*) and then find a neighboring third tile of the same color and shape that can be matched by making a swap (*find match*). This introduces an additional cost to visual search because of a memory search for multiple target categories [5,6,51].

### Research Questions

The goal of this study was to develop and evaluate the feasibility of a TMM3 game-based visual search assessment task, called search and match task (SMT), for older adults with and without cognitive impairment. To this purpose, we combined a TMM3 puzzle video game with the visual search paradigm. The SMT controls variables that affect visual search performance and supports the collection of search time data. To control variables that affect visual search performance, difficulty levels were created by manipulating the width and height of the puzzle board (set size) and the number of different types of tiles (distractor heterogeneity). In addition, the SMT was designed as a single-target visual search task with multiple target categories. A preliminary user study in young, older, and oldest adults was conducted to preliminarily evaluate the SMT.

First (hypothesis 1), we expected that with increasing the total number of items (set size) and decreasing the number of different types of tiles (distractor heterogeneity), the task difficulty increases and vice versa. An increase in task difficulty is hypothesized to result in longer search times and higher numbers of errors (false moves) [49,52]. Second (hypothesis 2), we expected the performance on the SMT to be significantly influenced by age. On the basis of previous literature that showed age-related declines in inefficient visual search [21] and performance on a commercial TMM3 video game [5], we expected young adults to perform better than older adults and older adults to perform better than oldest adults. Third (hypothesis 3), as previous studies have suggested, we expected an association between performance on the SMT and assessments for global cognitive ability and cognitive functions required to play the SMT. These include measures of selective and divided attention [41], visual search [5,6], and spatial processing speed and pattern recognition [6,49].

## Methods

### Participants

In total, 28 healthy younger (20 female and 8 male) aged between 18 and 31 years (mean 21.68 years, SD 2.86), 13 healthy older adults (7 female and 6 male) aged between 64 and 79 years (mean 70.54 years, SD 3.82), and 11 oldest adults (9 female and 2 male) aged between 86 and 94 years (mean 89.27, SD 3.29) participated in this study. The younger adults were recruited from the University of Bern student participant pool, older adults were recruited from the Seniors University of Bern, and oldest adults were recruited through seniors’ residences in Olten and Bern, Switzerland. The exclusion criteria for participation were (1) insufficient coordinative, motor, and perceptual ability to handle a tablet computer and (2) history of neurological or psychiatric deficits. All participants had normal or corrected-to-normal vision. All participants provided written informed consent in accordance with the latest version of the Declaration of Helsinki before participation. The cantonal ethics committees of Bern and Northwest and Central Switzerland granted the ethics approval for this study.

### Neuropsychological Assessment

A total of 5 neuropsychological tasks were used to assess the concurrent criterion validity of the SMT. The trail-making test (TMT) [53] was used as a paper-and-pencil measure of attentional function. The TMT trails A measures selective attention, visual scanning, and visuomotor processing, whereas the TMT trails B measures divided attention, working memory, and inhibition [53,54]. Visual search performance was assessed with the *visual scanning* subtest from the computerized test of attentional performance (TAP) [55,56] that is used as a screening measure for visual attention [57]. In this task, participants actively scanned a 5×5 matrix and indicated whether a specific target stimulus (square with top opening) was present or not among 3 types of similar distractor stimuli (squares with openings on the left, right, or bottom). The pattern comparison task (PCT) [58] was used as a measure of spatial processing speed and pattern recognition ability. The PCT requires participants to examine a pair of 8-dot patterns shown on the left and right half of the screen and determine whether they are similar or different. The Montreal Cognitive Assessment (MoCA) [59] was administered as a measure of global cognitive ability.

### Task Perception and Usability Assessment

Subjective acceptance of the SMT was assessed with the Perception of Game Training Questionnaire [60]. In this questionnaire, participants rated the extent to which they found playing the SMT *enjoyable*, *challenging*, and *frustrating* as well as their motivation while playing the mazes on a 7-point Likert scale. The 10-item system usability scale (SUS) was used to measure user experience, usability, and learnability of the SMT. The SUS provides a composite score from 0 to 100, where a higher number indicates a higher usability [61].

### Characteristics and Development of the Search and Match Task

#### Search and Match Task Description

The SMT was designed as an experimentally controlled pattern-matching visual search task that combines advantages from both computerized visual search tasks and puzzle video games. The SMT is played on a grid-based puzzle board with a given set size (width × height) that is randomly filled with tiles from a set of uniquely colored shapes (tiles) on a gray background (see Figure 3). The SMT provides a total of 71 difficulty levels, where each level is defined by a combination of set size of the board and the number of sets of tiles.

Each difficulty level in the SMT comprises trials with 4 single-target moves. For each trial, the goal is to look for a target pattern on the puzzle board (*search*) and make a move to horizontal or vertical sequence of 3 identical tiles (*match*; see Figure 3). Moves are performed by swapping the position of 2 adjacent tiles in any of the 4 cardinal directions using the mouse or a touch-sensitive screen. A move is only valid when it creates a match. Invalid moves are not allowed, and the swapped tiles will bounce back to their initial place. After valid moves, tiles above the matched tiles fall into the now empty cells, and the resulting empty cells at the top of the board are filled with new tiles [48,52]. Therefore, to finish a difficulty-level trial, participants must make 4 consecutive matches (see Figure 3, *Game*).

In TMM3 puzzle games, there are multiple potential matches on the puzzle board at a time and search difficulty depends on the number of potential matches present on a puzzle board [6]. To study the effects of the manipulated difficulty variables in a controlled manner, the SMT was designed as a single-target search task. SMT trials are self-terminating and end as soon as the single target pattern on the puzzle board has been found and matched by making a valid move (see Figure 3, *Game*).

#### Search and Match Task Difficulty Parameters and Development

A full factorial analysis was used to generate multiple difficulty levels for the SMT [62,63]. Difficulty levels were generated by constructing restricted combinations of width (w) and height (h) of the puzzle board (set size), and the number of tile types (t) varied over a range from 4 to 8 [49,52].

First, all possible combinations of puzzle board widths and heights from 4 to 8 were generated: (w, h) = {4, 5, 6, 7, 8} × {4, 5, 6, 7, 8} = 25 (see Figure 3, *Set size*). The puzzle board size determines the total number of tiles on the puzzle board that must be checked to find a target pattern configuration of tiles. With increasing set size, the time to find a target pattern increases (*set size effect*) [5,6,50]. Second, for each puzzle board size, the number of tile types (t) was set from 4 to the maximal value of height or width of the puzzle board. This resulted in 95 difficulty levels: (w, h, t) = {4, 5, 6, 7, 8} × {4, 5, 6, 7, 8} × {4 ≤ t ≤ max(w, h)} (see Figure 3, *Distractor heterogeneity*). Tile types were 8 regular convex polygons (3 to 11 sides) with a unique color. The number of tile types affects the number of tiles on the puzzle board that are identical to the tiles that form the target pattern (*sharing*) and the number of tiles that do not (*grouping*). More tile types increase grouping and make it easier to find a target pattern [49,64].

Third, playable trials were generated for each of the 95 task difficulty levels using a brute force–like algorithm programmed in Python (see Multimedia Appendix 1. The SMT was specifically designed such that all puzzle boards within a trial of 4 successive matches contain exactly 1 single-target pattern. To achieve this, the algorithm first generated a 2-dimensional array (width × height), randomly filled with tiles from a range of number of tile types (tile types) for each level. The algorithm checked whether the board contained exactly 1 target pattern (see Figure 3, *Game*, *search*) and solved it and repeated checking for 1 target pattern only (see Figure 3, *Game*, *match* and *fill*). When this process could be recursively performed 4 times in a row, it was considered a playable trial (see Figure 3, *Game*). From the 95 task difficulty levels, all levels with a minimum of 47 playable trials were selected and sorted by set size. This step yielded playable trials for 71 of the 95 prespecified difficulty levels (see Multimedia Appendix 1). The 71 generated difficulty levels were then divided into 2 parallel versions with 40 difficulty levels, each based on set size (see Multimedia Appendix 1). Both parallel versions contained all available square (w=h) difficulty levels, whereas the rectangular (w ≠ h) difficulty levels were assigned to the 2 versions in a parallelized fashion. This way, the number of levels could be reduced, while providing all available set sizes (w × h).

### Procedure

First, participants were informed about the procedures of the user study and written consent was obtained. Second, in the cognitive assessment session, the MoCA and the TMT trail A and trail B (completed by all participants) were administered in paper-pencil format, whereas the computerized visual scanning TAP task and the PCT (completed only by young and older adults) were presented on a computer. Third, in the difficulty evaluation session, participants played the pregenerated SMT difficulty levels. The SMT visual search task was played on a tablet computer (Apple 12.9” iPad Pro, Apple Inc) with a version of the SMT programmed in Unity 3D (Unity Technologies). To ensure that participants understood how to play the SMT, they were first provided instructions (see Instructions and Task in the Multimedia Appendix 2 and ) and a practice block. The experimenter read the instructions to the participants and showed them the 3 basic target patterns to look out for. In addition, the participants were told that there was only 1 target pattern to match at a time. After that, the participants played a practice block with 3 incremental difficulty levels (w, h, p) = {(4, 4, 4), (5, 5, 5), (6, 6, 6). Here, they were shown how to use the *hint* button that highlights the target pattern when they could not find it and encouraged to use it when needed. In the test block, participants completed the SMT difficulty levels. The younger and older adults completed the full set of difficulty levels (long version, 40 levels) of the SMT. On the basis of previous experience with oldest adults, we chose to use a shortened version of difficulty levels with the lower third of difficulty levels (short version, 12 levels; see Multimedia Appendix 1 and ). This was mainly for reasons of time and not to overburden the participants. The 2 parallel versions of the task were counterbalanced across participants. For each difficulty level, a trial was randomly drawn from the respective difficulty level folder of pregenerated trials. Each trial for every difficulty level consisted of 4 consecutive matches (Figure 3, *Game*). After completing each level, participants were asked to rate the difficulty of the played trial on a 10-point Likert scale ranging from 1 (*very easy*) to 10 (*very difficult*). The difficulty levels in the test block were presented in random order to avoid learning effects that might occur when presented in incremental order [65]. After completing all levels, the participants evaluated the usability and their experience with the SMT by filling in the SUS and the Perception of Game Training Questionnaire.

### Statistical Analysis

A summary file with entries for each played move on the SMT was stored. Each move entry included the trial number, height and width of the puzzle board, and the number of unique tile types. Furthermore, the move number, time to make the move (search time), accuracy (correct or false move), and whether a hint was used to make a correct move were recorded. To calculate the search time for each puzzle board, all false moves leading up to a correct move were summed up. Trials with outliers in search time (search times greater or less than 1.5 × interquartile range for each age group) were removed from analysis. The following time-based performance indicators were calculated: overall solving time (min), average target search time (sec), average processing time per item (sec), and search slope (sec/item).

Processing time per item was calculated by dividing search time by the number of items in the display.

Search slope was calculated by means of a general linear model (GLM), assuming gamma distribution because of nonnormal search time data. The model included search time as a response variable and an interaction term for set size and age group as a predictor variable. Error-based performance measures included the number of false moves and the number of used hints. For all further analyses, trials where a hint was used were excluded.

First, age-group differences in demographic variables, neuropsychological test measures, and SMT puzzle game performance measures were analyzed. Visual inspection of histograms, quantile-quantile plots, and Shapiro-Wilk and Anderson-Darling (for the long puzzle version data) tests revealed that these variables were nonnormally distributed. Statistical differences between the 3 age groups were performed in R Version 1.1.463 [66] using the nonparametric Kruskal-Wallis test, with subsequent pairwise Wilcoxon rank sum tests (using Bonferroni correction) for post hoc intergroup comparisons. An alpha value of .05 was used to determine significance. Post hoc comparisons between search slopes by age group were performed using Tukey's honest significance test implemented in the lstrends function from the lsmeans package [67] for R.

For all analyses below, only search times for trials without hints were analyzed.

Second, the effect of the 2 manipulated task parameters (as per hypothesis 1) and age (as per hypothesis 2) on search time (dependent variable) was tested. As the search time data were positively skewed (short version dataset: skewness=0.65, SD 1.11; long version dataset: skewness=0.56, SD 0.61), we performed a general linear mixed-effect model (GLMEM) analysis using the lme4 package [68]. To approximate the distribution of the search time data, we assumed a gamma distribution with inverse link function (see the study by Lo and Andrew [69] for recent guidelines). In addition, 2 GLMEMs assuming gamma distribution (inverse link function) with search time as outcome; set size, the number of unique tile types (within-subjects factors), and age (between-subjects factor) as fixed effects; and a random intercept per subject as a random effect were fitted. We performed this analysis separately on the short puzzle difficulty version (12 levels), which was played by all age groups, and on the long or full puzzle difficulty version (40 levels, including the 12 levels from the short version), which was played by the young and older adults.

Third, external validity was examined through correlation analyses (using the Spearman rank correlation coefficients) between the geometric mean search time and the performance on cognitive tests with measures of selective (TMT A completion time) and divided (TMT B completion time), visuospatial processing speed and pattern recognition (mean overall response time), and visual search (mean response time for target present trials). Separate partial correlation analyses (again using the Spearman rank correlation coefficients), controlling for the effect of participant age, were performed. Both analyses were performed separately for the short (all age groups) and long (young and older adults) difficulty-level versions using the sjstats [70] and ppcor package [71].

## Results

### Results for Demographic Variables and Neuropsychological Tests

Demographic variables and neurocognitive measures by age group are shown in Table 1. Regarding demographic characteristics, the 3 age groups differed significantly on age at test (*χ*^2^_2_=42; *P*<.001) and years of education (*χ*^2^_2_=16; *P*<.001). The oldest adults (mean 89.27) were significantly older than the older adults (mean 70.54; *P*<.001) and young adults (mean 21.68; *P*<.001), and the older adults were significantly older than the young adults (*P*<.01). Duration of education was significantly lower in the oldest adults (mean 11.73) group compared with the older (mean 15.92; *P*<.01) and young (mean 14.47; *P*<.01) adults, but it was not different between older and young adults (*P*=.53).

Concerning neuropsychological test measures, global cognitive ability was significantly different between the 3 groups (*χ*^2^_2_=14.5; *P*<.001) and significantly lower in the oldest adults (mean 24.27) compared with the young adults (mean 28.32; *P*<.001). In terms of performance on attentional tasks, there were significant effects of age group in selective attention time (*χ*^2^_2_=15.2; *P*<.001) but not errors (*χ*^2^_2_=3.3; *P*=.19) and in divided attention time (*χ*^2^_2_=26.6; *P*<.001) as well as errors (*χ*^2^_2_=8.9; *P*<.01). In selective attention, the younger adults (mean 26.44) were significantly faster than older adults (mean 36.15; *P*=.027) and oldest adults (mean 58.18; *P*<.001), and the older adults were significantly faster than the oldest adults (*P*<.01). In divided attention, younger adults (mean 50.78) were significantly faster than older adults (mean 107.69; *P*<.001) and oldest adults (mean 155.60; *P*<.001). Younger adults (mean 0.50) made significantly fewer errors than older (mean 2.85; *P*=.04) and oldest adults (mean 1.55; *P*=.02).

In the computerized visual search and visuospatial processing task that was completed only by the younger and old adult group, there was a significant difference between younger and older adults in visual search (trials with target present: mean 2.02 vs 3.85; *χ*^2^_1_=21.1; *P*<.001 and trials with targets absent: mean 3.62 vs mean 6.96; *χ*^2^_1_=19.6; *P*<.001) and visuospatial processing (mean 1.69 vs mean 3.85; *χ*^2^_1_=22.5; *P*<.001).

**Table 1:** Means and SDs for the demographic variables and neuropsychological test measures by age group.

Variables		Young adults aged 18-35 years (n=28)	Older adults aged 65-85 years (n=13)	Oldest adults aged 85+ years (n=11)	*P* value	Group comparison
**Demographic variables**						
	Age (years), mean (SD)	21.68 (2.86)	70.54 (3.82)	89.27 (3.29)	<.001^a^	Young < older < oldest
	Gender (female/male)	20/8	7/6	9/2	—^b^	—
	Education (years), mean (SD)	14.47 (1.94)	15.92 (2.42)	11.73 (1.49)	<.001^a^	Young, older < oldest
**Neuropsychological tasks, mean (SD)**						
	Montreal cognitive assessment^c^ total	28.32 (1.74)	27.15 (3.08)	24.27 (3.04)	<.001^a^	Young > oldest
	TMT-A^d^ time (seconds)	26.44 (9.38)	36.15 (7.69)	58.18 (15.54)	<.001^a^	Young < older < oldest
	TMT-A errors, mean (SD)	0.00 (0.00)	0.08 (0.28)	0.18 (0.40)	.19^e^	NS^f^
	TMT-B^g^ time (seconds)	50.78 (19.30)	107.69 (43.21)	155.60 (49.72)	<.001^a^	Young < older, oldest
	TMT-B errors	0.50 (1.89)	2.85 (4.41)	1.55 (1.51)	.01^h^	Young < older, oldest
	Visual scanning TAP^i^ (seconds)	2.28 (0.56)	4.06 (0.64)	—	<.001^a^	Young < older
	Pattern comparison overall (sec)	1.63 (0.29)	2.97 (0.54)	—	<.001^a^	Young < older
**Task perception, average difficulty rating, and usability, mean (SD)**						
	Enjoyable^j^	5.32 (1.09)	5.92 (0.86)	6.10 (1.60)	.12^e^	NS
	Challenging^j^	4.00 (1.68)	5.62 (1.04)	5.50 (0.97)	.01^h^	Young < older
	Frustrating^j^	2.11 (1.45)	2.54 (1.61)	2.30 (2.21)	.74^e^	NS
	Motivating^j^	5.96 (1.07)	6.31 (0.48)	6.50 (0.97)	.25^e^	NS
	Average difficulty rating^k^ (short)	2.69 (1.62)	3.34 (1.48)	3.43 (2.24)	<.001^a^	Young < older, oldest
	Average difficulty rating^k^ (long)	2.82 (1.68)	3.43 (1.69)	—	<.001^a^	Young < older
	System Usability Scale score^l^	88.61 (7.28)	79.09 (15.50)	68.25 (18.78)	<.01^h^	Young > oldest

^a^Significant at the .001 level.

^b^Not applicable.

^c^Score: 1-30.

^d^TMT A: trail-making test, trail A.

^e^Significant at the .05 level.

^f^NS: not significant.

^g^TMT B: trail-making test, trail B.

^h^Significant at the .01 level.

^i^Visual scanning: subtest visual scanning from the computerized test of attentional performance.

^j^Perception of Game Training Questionnaire (7-point Likert scale).

^k^Single ease question (range: 1, *very easy* to 10, *very difficult*).

^l^Score: 0-100.

### Results for Perception, Average Difficulty Ratings, and Usability of the Search and Match Task

In terms of perception of the SMT as a game-based training, there were significant differences between the 3 age groups regarding ratings of challengingness (*χ*^2^_2_=10.2; *P*<.001). However, there were no significant differences in terms of enjoyment, frustration, and motivation while playing the SMT task. On the whole, young adults (mean 4.00) perceived the SMT as significantly less challenging than older adults (mean 5.62; *P*=.02).

Regarding average difficulty rating based on ratings for each difficulty level, there were significant age group differences in average difficulty ratings for both the short version played by the young, older, and oldest adults (*χ*^2^_2_=266.6; *P*<.001) and the long version (*χ*^2^_2_=479.4; *P*<.001) played by the young and older adults. Average difficulty ratings for all played levels in the short version revealed that younger adults rated these difficulty levels as significantly less difficult (mean 2.69) than both the older (mean 3.34; *P*<.001) and oldest adults (mean 3.43; *P*<.001). Average difficulty ratings for all levels in the long version further showed that the young adults (mean 2.82) gave significantly lower difficulty ratings than the older adults (mean 3.43; *P*<.001).

Overall system usability ratings for the SMT indicated a significant effect of age group (*χ*^2^_2_=10.4 *P*<.01). Oldest adults (mean 68.25) ranked the usability significantly lower than young adults (mean 88.61; *P*=.007). Individual usability ratings ranged from 72.50 (*good*) to 97.50 (*excellent*) in young, from 52.50 (*okay*) to 100 (*excellent*) in older, and from 32.50 (*unacceptable*) to 95.00 (*excellent*) in oldest adults [72].

### Performance on the Search and Match Task

For the short puzzle version, as shown in Table 2, time-based performance measures revealed significant age-group differences in overall completion time (*χ*^2^_2_=337.6; *P*<.001), average target search time for all trials (*χ*^2^_2_=374.1; *P*<.001), and trials without hints (*χ*^2^_2_=330.3; *P*<.001). Post hoc analyses for task completion time for the short difficulty level version showed that young adults (mean 5.34) were significantly faster compared with both older adults (mean 15.26; *P*<.001) and oldest adults (mean 21.99; *P*<.001). In addition, older adults were significantly faster than the oldest adults (*P*<.001).

**Table 2:** Means and SDs for search and match task performance measures by age group.

Variables		Young adults aged 18-35 years (n=28)	Older adults aged 65-85 years (n=13)	Oldest adults aged 85+ years (n=11)	*P* value	Group comparison
**Short puzzle version (w, h, t) = {4, 5, 6} × {4, 5, 6} × {4 ≤ t ≤ max(w, h)} = 12 levels**							
	Task completion time (min), mean (SD)	5.34 (1.91)	15.26 (6.23)	21.99 (9.02)	<.001^a^	Young < older < oldest	
	Average search time (seconds) with hints, mean (SD)	2.75 (1.58)	4.31 (3.86)	8.39 (7.99)	<.001^a^	Young < older < oldest	
	Average search time (seconds) without hints, mean (SD)	2.74 (1.57)	4.34 (3.91)	8.43 (8.05)	<.001^a^	Young < older < oldest	
	Search slope (sec/item)	1.37	.26	.49	.63^b^	Young = older = oldest	
	Processing time per item (sec), mean (SD)	0.11 (0.07)	0.27 (0.21)	0.42 (0.36)	<.001^a^	Young < older, oldest	
	Total number of false moves, mean (SD)	0.02 (0.14)	0.31 (0.46)	0.28 (0.45)	<.001^a^	Young < older, oldest	
	Total number of used hints, mean (SD)	0.15 (0.42)	0.35 (0.70)	0.61 (0.76)	<.001^a^	Young < older < oldest	
**Long puzzle version (w, h, t) = {4, 5, 6, 7, 8} × {4, 5, 6, 7, 8} × {4 ≤ t ≤ max(w, h)} = 40 Levels**							
	Task completion time, mean (SD)	17.75 (5.58)	35.94 (24.96)	—^c^	<.001^a^	Young < older	
	Average search time (seconds) with hints, mean (SD)	4.16 (5.19)	4.81 (4.30)	—	<.001^a^	Young < older	
	Average search time (seconds) without hints, mean (SD)	4.14 (5.21)	4.82 (4.34)	—	<.001^a^	Young < older	
	Search slope (sec/item)	1.69	−.56	—	<.001^a^	Young > older	
	Processing time per item (sec), mean (SD)	0.12 (0.16)	0.21 (0.18)	—	<.001^a^	Young < older	
	Total number of false moves, mean (SD)	0.02 (0.13)	0.28 (0.45)	—	<.001^a^	Young < older	
	Total number of used hints, mean (SD)	0.14 (0.42)	0.49 (0.88)	—	<.001^a^	Young < older	

^a^Significant at the .001 level.

^b^Not significant.

^c^Indicates long puzzle difficulty version not completed by oldest adults.

**Figure 4:**
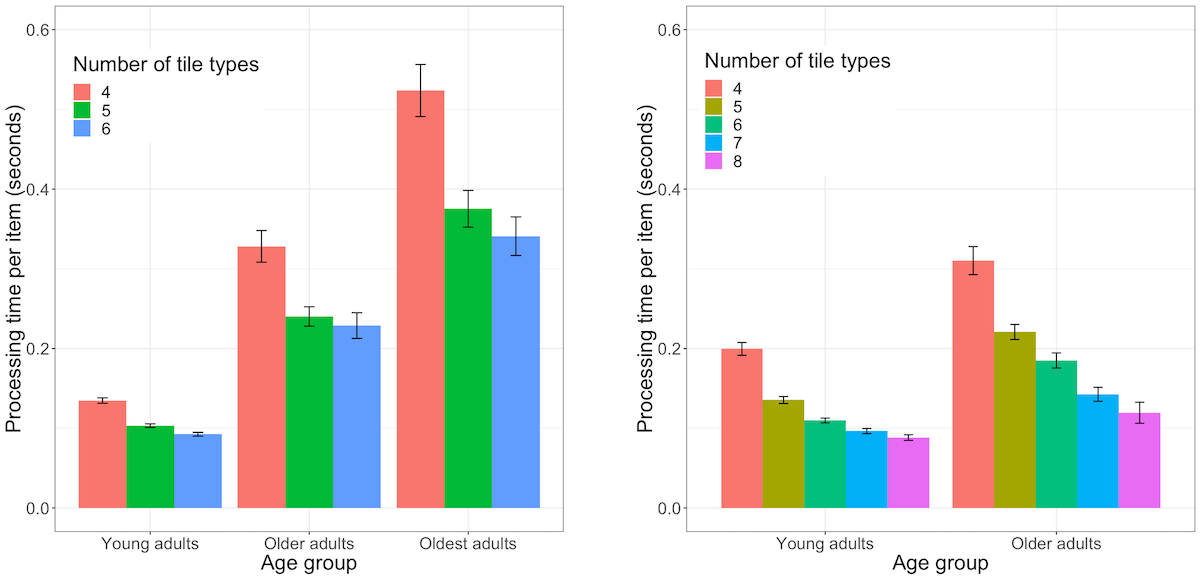
Average processing time per item (tile) for the short (left) and long (right) puzzle difficulty level version by age group and number of tile types.

Regarding average search time (for both trials where a hint was used and trials without hints), oldest adults (mean 8.39; mean 8.43) were significantly slower than both older adults (mean 4.32; mean 4.34; *P*<.001) and young adults (mean 2.75; mean 2.74, *P*<.001), and older adults were significantly slower than young adults (*P*<.001), respectively. In the analysis of search slopes for the short version puzzle difficulty levels, GLM analysis showed a significant effect of age group (*χ*^2^_2_=2113.1; *P*<.001) on search time. The effect of set size (*χ*^2^_2_=2.3; *P*=.13) and the interaction between set size and age group (*χ*^2^_2_=0.9; *P*=.63) were not significant. Post hoc analysis revealed that search slopes were not significantly different between young and older adults (*P*=.62), young and oldest adults (*P*=.69), and older and oldest adults (*P*=.96). Average processing time per item across different number of tile types revealed was significantly different between age groups (*χ*^2^_2_=705.7; *P*<.001). Younger adults (mean 0.15) took significantly less processing time per item than both older (mean 0.35; *P*<.001) and oldest (mean 0.61; *P*<.001) adults, and older than oldest adults (*P*<.001). Figure 4 (left) shows processing times per item by age group and by the number of tile types to illustrate the additional effect of distractor heterogeneity.

For the accuracy-based performance measures, there was a significant effect of age group on the total number of false (invalid) moves (*χ*^2^_2_=680.9; *P*<.001) and the total number of used hints (*χ*^2^_2_=563.8; *P*<.001). Compared with the young adults (mean 0.02), the older (mean 0.35; *P*<.001) and oldest adults (mean 0.28; *P*<.001) made significantly more false moves. The oldest adults (mean 0.61) used significantly more hints than both the older (mean 0.35; *P*<.001) and young adults (mean 0.15; *P*<.001), and the older adults significantly more than the younger adults (*P*<.001).

For the long puzzle version played by the young and older adults (Table 2), there was a significant effect of age group in overall task completion time (*χ*^2^_2_=698.9; *P*<.001), with older adults taking significantly longer than young adults to complete the long difficulty level version. For both trials with (*χ*^2^_2_=374.1; *P*<.001) and without hints (*χ*^2^_2_=81.7; *P*<.001), average search time was significantly slower in older than in young adults. The GLM analysis for search slopes revealed a significant interaction between age group and set size (*χ*^2^_2_=14.30; *P*<.001) and significant effects of set size (*χ*^2^_2_=21.2; *P*<.001) and age group (*χ*^2^_2_=123.6; *P*<.001) on search time. Post hoc comparisons showed that search slopes were significantly different between young and older adults (*P*<.001). In addition, age significantly influenced average processing time per item (*χ*^2^_2_=383.6; *P*<.001) and older adults (mean 0.21) were significantly slower than younger adults (mean 0.12; *P*<.001). Figure 4 (right) shows processing times per item by age group and separately for the different number of tile types to illustrate the effect of distractor heterogeneity. In terms of accuracy-based performance measures, older adults made significantly more false moves (*χ*^2^_2_=1770.9; *P*<.001) and used significantly more hints (*χ*^2^_2_=664; *P*<.001)) than young adults.

### Results for Generalized Linear Mixed-Effect Models

For the short version, as shown in Table 3, the GLMEM revealed a significant positive effect of set size (*F*_1,2760_=2.18; *P*=.01; Cohen f=.026), a significant negative effect of the number of tile types (*F*_1,2760_=8.17; *P*=.01; Cohen f=.05) and a significant positive effect of age (*F*_1,2760_=408.3; *P*<.001; Cohen f=.35) on target search time.

**Table 3:** General linear mixed-effect model results for the effect of set size, number of tile types (distractor heterogeneity), and age on search time on the short puzzle version with all age groups (young, old, and oldest adults) and the long puzzle version (young and older adults).

Variables	Short difficulty levels version, search time (seconds)			Long difficulty levels version, search time (seconds)		
	Estimates	CI	*P* value	Estimates	CI	*P* value
Set size	1.29	0.34 to 2.24	.01^a^	2.24	1.84 to 2.64	<.001^b^
Number of tile types	−8.87	−15.61 to −2.13	.01^a^	−16.46	−20.50 to −12.42	<.001^b^
Participant age	3.82	3.37 to 4.27	<.001^b^	1.90	1.19 to 2.61	<.001^a^

^a^Significant at the .01 level.

^b^Significant at the .001 level.

For the long version, the GLMEM analysis revealed a significant positive effect of set size (*F*_1,7569_=34.70; *P*<.001; Cohen f=.08), a significant negative effect of the number of tile types (*F*_1,7569_=35.86; *P*<.001; Cohen f=.081), and a significant positive effect of age (*F*_1,7569_=14.12; *P*<.001; Cohen f=.051) on target search time.

### Results for External Validity Testing

To assess external validity, time-based performance on the SMT (geometric mean search time) was compared with performance on standard neuropsychological tests. External validity testing results are reported separately for the short and long difficulty levels version in Table 4.

For the short puzzle difficulty level version, Spearman correlation analyses showed significant positive associations between geometric mean search time and TMT A completion time (*r*=.724; *P*<.001) and TMT B completion time (*r*=.755; *P*<.001). Furthermore, there was a significant negative correlation between geometric mean search time and the MoCA score (*r*=−.453; *P*=.01). To further evaluate the contribution of age on the neuropsychological tests, partial correlations of geometric mean search time with the neuropsychological test measures controlling for age were assessed. The partial correlation of both TMT A (*r*=.374; *P*=.02) and TMT B (*r*=.342; *P*=.03) completion time with geometric mean search time remained significant when controlling for age. However, the partial correlation between MoCA (controlling for age) and geometric mean search time was not significant (*r*=−.178; *P*=.27).

For the long puzzle level version, geometric mean search time was significantly positively associated with TMT A (*r*=.546; *P*<.001) and TMT B (*r*=.573; *P*=.001) completion time, average target search time on a computerized visual search task (*r*=.430; *P*=.007) and average response time on a visuospatial processing and pattern recognition task (*r*=.543; *P*<.001). However, the association with MoCA scores (*r*=−.223; *P*=.16) was not significant. When controlling for age, only the positive relationship with TMT A (*r*=.49; *P*=.008) and TMT B (*r*=.43; *P*=.02) completion time remained significant. However, the partial correlation between SMT performance and visual search task performance (*r*=.064; *P*=.75) as well as performance on the visuospatial processing and pattern recognition task (*r*=.038; *P*=.85) was not significant anymore.

**Table 4:** Correlations and partial correlations (controlling for age) between search and match task performance (geometric mean search time) and performance on neuropsychological tests for the short and long puzzle versions.

Test measure	Short difficulty level version, geometric mean search time				Long difficulty level version, geometric mean search time			
	Simple correlation		Adjusted for age		Simple correlation		Adjusted for age	
	ρ	*P*	ρ	*P*	ρ	*P*	ρ	*P*
Trail-making test A completion time	.724	<.001^a^	.373	.02^b^	0.546	.01^c^	.49	.01^c^
Trail-making test B completion time	.755	<.001^a^	.342	.03^b^	0.573	.01^c^	.43	.02^b^
Montreal cognitive assessment	−.435	.01^c^	−.178	.27^d^	−.223	.162^d^	−.316	0.1^d^
Pattern Comparison	—^e^	—	—	—	.543	<.001^a^	.064	.75^d^
Visual Scanning	—	—	—	—	0.43	.01^c^	.038	.2^d^

^a^Significant at the .001 level.

^b^Significant at the .05 level.

^c^Significant at the .01 level.

^d^Not significant.

^e^Ttests not completed by the oldest adults and therefore not used in correlational analysis across all age groups.

## Discussion

### Principal Findings

The aim of this study was to develop and examine the initial validity of an experimentally controlled version of a popular TMM3 puzzle video game—the SMT. The SMT was specifically designed as a single-target, pattern-matching visual search task with multiple levels of difficulty. The preliminary results of this study show that an entertaining commercial puzzle video game can be adapted as a visual search task that allows control over task difficulty and collection of relevant performance data.

First, preliminary results of our user study indicate that the SMT difficulty can be manipulated with 2 task parameters. Performance on the task (search time) increased with the total number of search items in the display (set size) and decreased with the number of types of tiles (distractor heterogeneity). Second, we found a significant effect of age group on search time in the SMT. Young adults had faster search times than older and oldest adults, and older adults were faster than oldest adults. Third, the SMT showed significant relationships with cognitive tests that measure general cognitive ability, selective and divided attention, and visuospatial and pattern recognition ability.

### Comparison With Previous Work

Unlike newer approaches that have introduced game-like elements to visual search tasks (*task gamification*), this study combines an entertaining and enjoyable puzzle video game with features of the visual search paradigm (*game taskification*). This approach achieves 3 goals. First, we make use of the motivational properties of a highly popular recreational puzzle game that engages visual search and other cognitive abilities and is user-friendly and enjoyable for older adults. Second, it allows researchers to control and systematically vary variables that affect the task difficulty. This allows to accommodate players with different levels of cognitive ability, which is particularly important for diagnostic and interventional purposes. Third, this supports collecting relevant performance data otherwise not available from computer games.

First, mixed-model analyses from the initial validation study indicate that the SMT meets the 2 criteria used to manipulate the difficulty levels of the task. The results revealed significant positive effects of the total number of search items in the display (set size) and a significant negative effect of the number of different types of distractors (distractor heterogeneity) on the performance in the SMT (search time).

The significant positive effect of set size on target search time is consistent with findings from previous visual search literature. Set size effects are well documented and determine task difficulty in feature conjunction and configuration search tasks. More recently, set size manipulations have been successfully used in computerized visual search training tasks to adapt the difficulty of the task [28,39,73]. Similarly, we conclude that finding a target pattern in the TMM3 game–based SMT becomes more difficult with increasing set size as it requires searching a larger puzzle board area with more tiles that may potentially constitute a target pattern. The significant negative effect of distractor heterogeneity on target search time suggests that finding a target pattern (*match*) is less difficult when there are more different types of tiles on the puzzle board. This indicates a facilitatory effect of the heterogeneity of the puzzle board. When the number of tiles and heterogeneity increase, there are fewer tiles that share the visual feature with the target pattern (*grouping effect*) and pattern detection is easier. Conversely, when heterogeneity decreases, more tiles are identical to the tiles that constitute a target pattern (*sharing effect*). This might have a distracting effect and pattern detection is harder [49,64].

Second, the age effect seen in our study agrees with similar findings from conjunction and spatial configuration search literature that reported age-related declines in search performance that start in middle-aged adults and progress throughout older age [21,74]. Moreover, we validate a recent study that found a significant association between age and game-based high scores (ie, number of matched tiles) on a classical TMM3 puzzle game in a similar sample of young and older adults [5]. Instead of match-based high scores, our study provided search time– and error-based measures otherwise not available from commercial games. This finding reflects the interindividual variability and differences in visual search ability seen in normal aging but also in neurodegenerative diseases and after brain injury. This variability should be addressed with tools providing multiple difficulty levels, in particular for diagnostic and interventional purposes [17,28]. As a puzzle game–based visual search task, the SMT provides a range of difficulty levels aimed to accommodate the needs for clinical settings that are usually not met by commercial puzzle video games.

Third, external validity testing provides preliminary support for a TMM3 puzzle game–based visual search task to engage perceptual and cognitive abilities that are subject to age-associated decline. Our results revealed significant associations between performance on the SMT (search time) with measures of selective and divided visual attention, visuospatial processing and pattern recognition, and visual search.

These findings are in agreement with findings from 2 earlier studies that reported significant relationships between performance on a TMM3 puzzle game and simple visual search tasks in younger and older adults [5] and measures of selective and divided attention in older adults [41,75]. Moreover, this relates the SMT to perceptual inhibition skills required to suppress distracting tiles when searching for a target pattern and working memory skills needed to keep track of multiple separate groups of tiles [6,73]. For both the young and older adults, we further found an association with visuospatial processing speed and pattern recognition. This underlines that the SMT requires higher-level pattern recognition ability to find the target patterns that can be matched [6,49].

Partial correlation analyses controlling for age revealed moderate significant positive correlations of performance on the SMT (both short and long version difficulty levels) with TMT A and TMT B completion time. This suggests that our TMM3 game–based task involves psychomotor processing, selective and divided visual attention, and executive components such as inhibition and updating. However, the correlations between game performance and global cognitive ability as well as computerized assessments of visual search and pattern comparison disappeared when age was controlled. A likely explanation for these findings could be found in the design of this study. The oldest adults only played the short difficulty level version that might not have been sufficient to capture changes in search efficiency as shown in the absence of an age difference in search slopes. In addition, the younger and older adults who played the long difficulty level version and performed the computerized visual search and PCT did not differ in global cognitive ability.

Finally, our results on perception and usability of the SMT replicate 2 previous studies [42,43] that showed that TMM3 puzzle games are enjoyable, motivating, user-friendly, and easy to use. As another study showed, older adults also value the perceived benefits (ie, improving cognition and stress relief) of this game [43]. However, we found that the perception of overall challengingness and level-based difficulty ratings differed between age groups. On the one hand, this might simply reflect the age-related differences in time- and error-based performance measures on the SMT. On the other hand, it might be that it was harder for older adults to learn to play the game.

### Limitations

This preliminary study included a small sample size of individuals with age-appropriate cognitive ability. Therefore, more research is needed using larger samples across a wider range of age groups and cognitive abilities. Although convergent validity indicates promising relationships with measures of attention, visuospatial, and executive function, a wider range of cognitive tasks is required to more comprehensively establish the relationship between the puzzle game–based visual search task and neuropsychological tests of cognitive functions. This would help better determine the validity of puzzle game–based tasks for assessing and monitoring age-related declines in visual search and other cognitive abilities.

Furthermore, this initial validation study is cross-sectional, and participants played age-appropriate sets of difficulty levels for the game-based visual search task only once. Therefore, older and oldest adults played the game for the first time and this task novelty might have affected task performance. This is reflected by the fact that the game was perceived as more challenging and difficult by the older and oldest adults. A reason for this is that participants need to memorize the basic target pattern configurations that must be searched and matched in order to play the game [76]. It is likely that older adults not only were slower in finding the target per se but also found it harder to memorize and learn the actual different target patterns. In addition, the SMT was designed as a single-target visual search task. Compared with commercial TMM3 puzzle games with multiple targets, this might additionally make the SMT harder to play. In future studies, this should be addressed by looking at learning effects when playing the SMT over longer time periods.

Our results indicate that the SMT difficulty can be successfully varied by manipulating set size and distractor heterogeneity. However, there are potential factors that we did not control that might additionally influence the difficulty of the SMT. First, unfortunately, we were not able to generate playable trials for the full range of the 95 difficulty levels specified in the full-factorial analysis (see Multimedia Appendix 1). These missing levels particularly concern levels with larger set sizes and small number of types of tiles: for example, (w, h, t) = 8, 8, 4. Owing to the low number of tile types, there were too many target patterns per puzzle board, such that is was impossible to generate 4 consecutive matches with only 1 target pattern per puzzle board. This of course limits the fine-grained range of difficulty levels we intended to provide. Second, the type and location of the target in the SMT was not controlled and left to random. Thus, we could not control the potential effects of different target types and target location as well as the effect of distance between targets across consecutive matches. Finally, this study used a restricted range of difficulty levels for reasons of time and burden. However, the algorithm used to generate the SMT difficulty levels in this study allows to generate more exhaustive levels for future studies.

### Conclusions

Taken together, this study shows that an everyday puzzle game–based task can be experimentally controlled and provides relevant performance data to assess visual search and cognitive abilities in normal aging. The game-based SMT is enjoyable, motivating, and user-friendly for older adults.

Future studies might also use the potential of such *taskified* or *hybrid* games to assess whether they can reliably assess cognitive abilities and impairment in older adults and patients with dementia or brain injury. This would help better determine the validity of puzzle game–based tasks for assessing and monitoring age-related declines in visual search and other cognitive abilities. Finally, the potential of an intervention using the available difficulty levels to practice visual search and cognitive ability in an enjoyable and adaptive way could be further explored.

## Appendix

Multimedia Appendix 1 Lists of generated puzzle levels as referenced in manuscript text.

Multimedia Appendix 2 Instructions for the search and match mask Windows.

Multimedia Appendix 3 Instructions for the search and match task Mac OS.

Multimedia Appendix 4 Search and match task for Windows.

Multimedia Appendix 5 Search and match task for Mac OS.

## Abbreviations

AD: Alzheimer’s disease
GLM: general linear model
GLMEM: general linear mixed-effect model
MCI: mild cognitive impairment
MoCA: Montreal cognitive assessment
PCT: pattern comparison task
RT: reaction time
SMT: search and match task
SUS: System Usability Scale
TAP: test of attentional performance
TMM3: tile-matching match-3
TMT: trail-making test

## References

[1] Horowitz TS (2017). Prevalence in visual search: from the clinic to the lab and back again. Jpn Psychol Res.

[2] Wolfe J, Wixted JT (2018). Visual search. Stevens' Handbook of Experimental Psychology and Cognitive Neuroscience.

[3] Cohen R, Cohen R (2014). Attention disturbances associated with neurological disease. The Neuropsychology of Attention.

[4] Kramer A F, Kray J, Bialystok E, Craik F (2006). Lifespan Cognition: Mechanisms of Change.

[5] Stroud MJ, Whitbourne SK (2015). Casual video games as training tools for attentional processes in everyday life. Cyberpsychol Behav Soc Netw.

[6] Oei AC, Patterson MD (2013). Enhancing cognition with video games: a multiple game training study. PLoS One.

[7] Chan LKH, Hayward WG (2013). Visual search. Wiley Interdiscip Rev Cogn Sci.

[8] Lorenzo-López L, Amenedo E, Cadaveira F, Maestú F, Gariépy Q, Ménard R (2010). Age-related changes in visual search mechanisms: a perspective from cognitive neuroscience. Handbook of Cognitive Aging: Causes, Processes and Effects.

[9] Horowitz S, Goldstein E (2010). Visual search. Encyclopedia of Perception.

[10] Horowitz T, Goldstein EB (2010). Visual search. Encyclopedia of Perception.

[11] Lester B, Vatterott D, Vecera S, Rizzo M, Anderson S, Fritzsch B (2018). Attention processing speed. The Wiley Handbook on the Aging Mind and Brain.

[12] Peelen MV, Kastner S (2014). Attention in the real world: toward understanding its neural basis. Trends Cogn Sci.

[13] Bruce N, Tsotsos J, Paletta L, Rome E (2008). An information theoretic model of saliency visual search. Attention in Cognitive Systems Theories and Systems from an Interdisciplinary Viewpoint.

[14] Huang L, Mo L, Li Y (2012). Measuring the interrelations among multiple paradigms of visual attention: an individual differences approach. J Exp Psychol Hum Percept Perform.

[15] Zanto T, Gazzaley A, Nobre AC, Kastner S (2014). Attention and ageing. The Oxford Handbook of Attention.

[16] Horowitz TS, Choi WY, Horvitz JC, Côté LJ, Mangels JA (2006). Visual search deficits in Parkinson's disease are attenuated by bottom-up target salience and top-down information. Neuropsychologia.

[17] Tales A, Haworth J, Nelson S, Snowden RJ, Wilcock G (2005). Abnormal visual search in mild cognitive impairment and Alzheimer's disease. Neurocase.

[18] Vallejo V, Cazzoli D, Rampa L, Zito GA, Feuerstein F, Gruber N, Müri RM, Mosimann UP, Nef T (2016). Effects of Alzheimer's disease on visual target detection: a "Peripheral Bias". Front Aging Neurosci.

[19] Callaghan E, Holland C, Kessler K (2017). Age-related changes in the ability to switch between temporal and spatial attention. Front Aging Neurosci.

[20] Kramer AF, Boot WR, McCarley JS, Peterson MS, Colcombe A, Scialfa CT (2006). Aging, memory and visual search. Acta Psychol (Amst).

[21] Potter LM, Grealy MA, Elliott MA, Andrés P (2012). Aging and performance on an everyday-based visual search task. Acta Psychol (Amst).

[22] McLaughlin PM, Borrie MJ, Murtha SJ (2010). Shifting efficacy, distribution of attention and controlled processing in two subtypes of mild cognitive impairment: response time performance and intraindividual variability on a visual search task. Neurocase.

[23] Tales A, Butler SR, Fossey J, Gilchrist ID, Jones RW, Troscianko T (2002). Visual search in Alzheimer's disease: a deficiency in processing conjunctions of features. Neuropsychologia.

[24] Tales A, Bayer AJ, Haworth J, Snowden RJ, Philips M, Wilcock G (2011). Visual search in mild cognitive impairment: a longitudinal study. J Alzheimers Dis.

[25] Landy KM, Salmon DP, Filoteo JV, Heindel WC, Galasko D, Hamilton JM (2015). Visual search in Dementia with Lewy Bodies and Alzheimer's disease. Cortex.

[26] Porter G, Leonards U, Wilcock G, Haworth J, Troscianko T, Tales A (2010). New insights into feature and conjunction search: II. Evidence from Alzheimer's disease. Cortex.

[27] Schmitter-Edgecombe M, Robertson K (2015). Recovery of visual search following moderate to severe traumatic brain injury. J Clin Exp Neuropsychol.

[28] Erez AB, Katz N, Ring H, Soroker N (2009). Assessment of spatial neglect using computerised feature and conjunction visual search tasks. Neuropsychol Rehabil.

[29] Ramzaoui H, Faure S, Spotorno S (2018). Alzheimer's disease, visual search, and instrumental activities of daily living: a review and a new perspective on attention and eye movements. J Alzheimers Dis.

[30] Reavis EA, Frank SM, Tse PU (2018). Learning efficient visual search for stimuli containing diagnostic spatial configurations and color-shape conjunctions. Atten Percept Psychophys.

[31] Yashar A, Carrasco M (2016). Rapid and long-lasting learning of feature binding. Cognition.

[32] Frank SM, Reavis EA, Tse PU, Greenlee MW (2014). Neural mechanisms of feature conjunction learning: enduring changes in occipital cortex after a week of training. Hum Brain Mapp.

[33] Leonards U, Rettenbach R, Nase G, Sireteanu R (2002). Perceptual learning of highly demanding visual search tasks. Vision Res.

[34] Scialfa CT, Jenkins L, Hamaluk E, Skaloud P (2000). Aging and the development of automaticity in conjunction search. J Gerontol B Psychol Sci Soc Sci.

[35] Chun M, Wolfe J, Robertson L (2012). Perceptual learning memory in visual search. From Perception to Consciousness: Searching with Anne Treisman.

[36] Sireteanu R, Rettenbach R (2000). Perceptual learning in visual search generalizes over tasks, locations, and eyes. Vision Res.

[37] Haim Erez Ab, Soroker N, Katz N (2018). Phasic alerting combined with visual spatial training: a novel therapeutic approach for unilateral spatial neglect. IPMRJ.

[38] Kennerly D, Ahroni B, Kaluszka A (2015). Google Patents.

[39] Waddington J, Linehan C, Gerling K, Williams C, Robson L, Hodgson T (2018). Evaluation of Eyelander: a video game designed to engage children and young people with homonymous visual field loss in compensatory training. J Vis Impair Blind.

[40] Ellenberg SR (2013). Semantic Scholar.

[41] Styron R (2015). A Pilot Study of the Effectiveness of Casual Video Games in Improving Cognition in People Aged 50 Years and Older.

[42] Chesham A, Wyss P, Müri RM, Mosimann UP, Nef T (2017). What older people like to play: genre preferences and acceptance of casual games. JMIR Serious Games.

[43] Whitbourne SK, Ellenberg S, Akimoto K (2013). Reasons for playing casual video games and perceived benefits among adults 18 to 80 years old. Cyberpsychol Behav Soc Netw.

[44] Thompson O, Barrett S, Patterson C, Craig D (2012). Examining the neurocognitive validity of commercially available, smartphone-based puzzle games. Psychol.

[45] Washburn DA (2003). The games psychologists play (and the data they provide). Behav Res Methods Instrum Comput.

[46] Anguera JA, Gazzaley A (2015). Video games, cognitive exercises, and the enhancement of cognitive abilities. Curr Opin Behav Sci.

[47] Omori M, Felinto A (2012). Analysis of motivational elements of social games: a puzzle match 3-games study case. Int J Comput Games Technol.

[48] Williams A (2017). History of Digital Games: Developments in Art, Design and Interaction.

[49] Gutwin C, Rooke C, Cockburn A, Mandryk R, Lafreniere B (2016). Peak-End Effects on Player Experience in Casual Games.

[50] Kitchell L, Parada F, Emerick B, Busey T (2001). Feature Selection Strategies Perceptual Expertise in Configuration Search Tasks.

[51] Biggs AT (2017). Getting satisfied with. Atten Percept Psychophys.

[52] Hadar D, Oren S (2015). School of Computer Science & Engineering.

[53] Schretlen D, Bobholz J, Brandt J (1996). Taylor & Francis.

[54] Strauss E, Sherman EM, Spreen O (2006). A Compendium Of Neuropsychological Tests: Administration, Norms, And Commentary.

[55] Zimmermann P, Fimm B Psytest.

[56] Zimmermann P, Fimm FB, Leclercq M, Zimmermann P, an Zomeren A (2002). A test battery for attentional performance. Applied Neuropsychology of Attention.

[57] Büttner G, Schmidt-Atzert L (2004). [Diagnosis of Concentration and Attention].

[58] Englund C, Reeves D, Shingledecker C, Thorne D, Wilson K DTIC Online.

[59] Nasreddine ZS, Phillips NA, Bédirian V, Charbonneau S, Whitehead V, Collin I, Cummings JL, Chertkow H (2005). The Montreal Cognitive Assessment, MoCA: a brief screening tool for mild cognitive impairment. J Am Geriatr Soc.

[60] Boot WR, Champion M, Blakely DP, Wright T, Souders DJ, Charness N (2013). Video games as a means to reduce age-related cognitive decline: attitudes, compliance, and effectiveness. Front Psychol.

[61] Brooke J, Jordan P W, Thomas B, McClelland I L, Weerdmeester B (1996). SUS-a quick and dirty usability scale. Usability Evaluation In Industry.

[62] Fraser J, Katchabaw M, Mercer RE (2014). A methodological approach to identifying and quantifying video game difficulty factors. Entertain Comput.

[63] van Kreveld M, Loffler M, Mutser P (2015). Automated puzzle difficulty estimation. IEEE.

[64] Nordfang M, Wolfe JM (2014). Guided search for triple conjunctions. Atten Percept Psychophys.

[65] Waage Ó (2014). GitHub.

[66] (2018). R Foundation for Statistical Computing.

[67] Lenth RV (2016). Least-squares means: the R package ismeans. J Stat Software.

[68] Bates D, Mächler M, Bolker B, Walker S (2015). Fitting linear mixed-effects models using lme4. J Stat Soft.

[69] Lo S, Andrews S (2015). To transform or not to transform: using generalized linear mixed models to analyse reaction time data. Front Psychol.

[70] Lüdecke D (2019). The Comprehensive R Archive Network.

[71] Kim S (2015). ppcor: an R package for a fast calculation to semi-partial correlation coefficients. CSAM.

[72] Bangor A, Kortum PT, Miller JT (2008). An empirical evaluation of the System Usability Scale. Int J Hum-Comput Interact.

[73] Richard's MM, Introzzi I, Zamora E, Vernucci S (2017). Analysis of internal and external validity criteria for a computerized visual search task: a pilot study. Appl Neuropsychol Child.

[74] Hommel B, Li KZ, Li S (2004). Visual search across the life span. Dev Psychol.

[75] Ellenberg S, Krauss S (2013). Semantic Scholar.

[76] Juul J, Keldorff R, Davidson D (2010). Depth in one minute: a conversation about bejeweled blitz. Well Played 2.0: Video Games, Value And Meaning.

