# Supplementary material for: Search and Match Task: Development of a Taskified Match-3 Puzzle Game to Assess and Practice Visual Search
Source: JMIR Serious Games. 2019 May 9;7(2):e13620. doi: 10.2196/13620 (PMC6532342; doi:10.2196/13620)
Supplement: Multimedia Appendix 1 [file games_v7i2e13620_app1.docx]

## Appendix A – Generated Search & Match Task Difficulty Levels

| **Level Number** | **Height** | **Width** | **Tile Types** | **Trials** |
| --- | --- | --- | --- | --- |
| 01 | 4 | 4 | 4 | 450 |
| 02 | 4 | 5 | 4 | 450 |
| 03 | 4 | 5 | 5 | 450 |
| 16 | 5 | 4 | 4 | 450 |
| 17 | 5 | 4 | 5 | 450 |
| 04 | 4 | 6 | 4 | 432 |
| 05 | 4 | 6 | 5 | 450 |
| 06 | 4 | 6 | 6 | 450 |
| 32 | 6 | 4 | 4 | 339 |
| 33 | 6 | 4 | 5 | 450 |
| 34 | 6 | 4 | 6 | 450 |
| 18 | 5 | 5 | 4 | 103 |
| 19 | 5 | 5 | 5 | 450 |
| 07 | 4 | 7 | 4 | 51 |
| 08 | 4 | 7 | 5 | 450 |
| 09 | 4 | 7 | 6 | 450 |
| 10 | 4 | 7 | 7 | 450 |
| 50 | 7 | 4 | 4 | 47 |
| 51 | 7 | 4 | 5 | 450 |
| 52 | 7 | 4 | 6 | 450 |
| 53 | 7 | 4 | 7 | 450 |
| 20 | 5 | 6 | 4 | 7 |
| 21 | 5 | 6 | 5 | 450 |
| 22 | 5 | 6 | 6 | 450 |
| 35 | 6 | 5 | 4 | 2 |
| 36 | 6 | 5 | 5 | 450 |
| 37 | 6 | 5 | 6 | 450 |
| 11 | 4 | 8 | 4 | 8 |
| 12 | 4 | 8 | 5 | 450 |
| 13 | 4 | 8 | 6 | 450 |
| 14 | 4 | 8 | 7 | 450 |
| 15 | 4 | 8 | 8 | 450 |
| 71 | 8 | 4 | 4 | 3 |
| 72 | 8 | 4 | 5 | 450 |
| 73 | 8 | 4 | 6 | 450 |
| 74 | 8 | 4 | 7 | 450 |
| 75 | 8 | 4 | 8 | 450 |
| 23 | 5 | 7 | 4 | 0 |
| 24 | 5 | 7 | 5 | 450 |
| 25 | 5 | 7 | 6 | 450 |
| 26 | 5 | 7 | 7 | 450 |
| 54 | 7 | 5 | 4 | 0 |
| 55 | 7 | 5 | 5 | 450 |
| 56 | 7 | 5 | 6 | 450 |
| 57 | 7 | 5 | 7 | 450 |
| 38 | 6 | 6 | 4 | 0 |
| 39 | 6 | 6 | 5 | 442 |
| 40 | 6 | 6 | 6 | 450 |
| 27 | 5 | 8 | 4 | 0 |
| 28 | 5 | 8 | 5 | 188 |
| 29 | 5 | 8 | 6 | 450 |
| 30 | 5 | 8 | 7 | 450 |
| 31 | 5 | 8 | 8 | 450 |
| 76 | 8 | 5 | 4 | 0 |
| 77 | 8 | 5 | 5 | 141 |
| 78 | 8 | 5 | 6 | 450 |
| 79 | 8 | 5 | 7 | 450 |
| 80 | 8 | 5 | 8 | 450 |
| 41 | 6 | 7 | 4 | 0 |
| 42 | 6 | 7 | 5 | 61 |
| 43 | 6 | 7 | 6 | 450 |
| 44 | 6 | 7 | 7 | 450 |
| 58 | 7 | 6 | 4 | 0 |
| 59 | 7 | 6 | 5 | 59 |
| 60 | 7 | 6 | 6 | 450 |
| 61 | 7 | 6 | 7 | 450 |
| 45 | 6 | 8 | 4 | 0 |
| 46 | 6 | 8 | 5 | 5 |
| 47 | 6 | 8 | 6 | 450 |
| 48 | 6 | 8 | 7 | 450 |
| 49 | 6 | 8 | 8 | 450 |
| 81 | 8 | 6 | 4 | 0 |
| 82 | 8 | 6 | 5 | 4 |
| 83 | 8 | 6 | 6 | 450 |
| 84 | 8 | 6 | 7 | 450 |
| 85 | 8 | 6 | 8 | 450 |
| 62 | 7 | 7 | 4 | 0 |
| 63 | 7 | 7 | 5 | 3 |
| 64 | 7 | 7 | 6 | 450 |
| 65 | 7 | 7 | 7 | 450 |
| 66 | 7 | 8 | 4 | 0 |
| 67 | 7 | 8 | 5 | 1 |
| 68 | 7 | 8 | 6 | 156 |
| 69 | 7 | 8 | 7 | 450 |
| 70 | 7 | 8 | 8 | 450 |
| 86 | 8 | 7 | 4 | 0 |
| 87 | 8 | 7 | 5 | 0 |
| 88 | 8 | 7 | 6 | 136 |
| 89 | 8 | 7 | 7 | 450 |
| 90 | 8 | 7 | 8 | 450 |
| 91 | 8 | 8 | 4 | 0 |
| 92 | 8 | 8 | 5 | 0 |
| 93 | 8 | 8 | 6 | 11 |
| 94 | 8 | 8 | 7 | 450 |
| 95 | 8 | 8 | 8 | 450 |

## Appendix B – Selected and Sorted Generated Search & Match Task Difficulty Levels

| **No** | **Level Number** | **Height** | **Width** | **Tile Types** | **Set Size** | **Trials** |
| --- | --- | --- | --- | --- | --- | --- |
| 1 | 01 | 4 | 4 | 4 | 16 | 450 |
| 2 | 02 | 4 | 5 | 4 | 20 | 450 |
| 3 | 03 | 4 | 5 | 5 | 20 | 450 |
| 4 | 16 | 5 | 4 | 4 | 20 | 450 |
| 5 | 17 | 5 | 4 | 5 | 20 | 450 |
| 6 | 04 | 4 | 6 | 4 | 24 | 432 |
| 7 | 05 | 4 | 6 | 5 | 24 | 450 |
| 8 | 06 | 4 | 6 | 6 | 24 | 450 |
| 9 | 32 | 6 | 4 | 4 | 24 | 339 |
| 10 | 33 | 6 | 4 | 5 | 24 | 450 |
| 11 | 34 | 6 | 4 | 6 | 24 | 450 |
| 12 | 18 | 5 | 5 | 4 | 25 | 103 |
| 13 | 19 | 5 | 5 | 5 | 25 | 450 |
| 14 | 07 | 4 | 7 | 4 | 28 | 51 |
| 15 | 08 | 4 | 7 | 5 | 28 | 450 |
| 16 | 09 | 4 | 7 | 6 | 28 | 450 |
| 17 | 10 | 4 | 7 | 7 | 28 | 450 |
| 18 | 50 | 7 | 4 | 4 | 28 | 47 |
| 19 | 51 | 7 | 4 | 5 | 28 | 450 |
| 20 | 52 | 7 | 4 | 6 | 28 | 450 |
| 21 | 53 | 7 | 4 | 7 | 28 | 450 |
| 22 | 21 | 5 | 6 | 5 | 30 | 450 |
| 23 | 22 | 5 | 6 | 6 | 30 | 450 |
| 24 | 36 | 6 | 5 | 5 | 30 | 450 |
| 25 | 37 | 6 | 5 | 6 | 30 | 450 |
| 26 | 12 | 4 | 8 | 5 | 32 | 450 |
| 27 | 13 | 4 | 8 | 6 | 32 | 450 |
| 28 | 14 | 4 | 8 | 7 | 32 | 450 |
| 29 | 15 | 4 | 8 | 8 | 32 | 450 |
| 30 | 72 | 8 | 4 | 5 | 32 | 450 |
| 31 | 73 | 8 | 4 | 6 | 32 | 450 |
| 32 | 74 | 8 | 4 | 7 | 32 | 450 |
| 33 | 75 | 8 | 4 | 8 | 32 | 450 |
| 34 | 24 | 5 | 7 | 5 | 35 | 450 |
| 35 | 25 | 5 | 7 | 6 | 35 | 450 |
| 36 | 26 | 5 | 7 | 7 | 35 | 450 |
| 37 | 55 | 7 | 5 | 5 | 35 | 450 |
| 38 | 56 | 7 | 5 | 6 | 35 | 450 |
| 39 | 57 | 7 | 5 | 7 | 35 | 450 |
| 40 | 39 | 6 | 6 | 5 | 36 | 442 |
| 41 | 40 | 6 | 6 | 6 | 36 | 450 |
| 42 | 28 | 5 | 8 | 5 | 40 | 188 |
| 43 | 29 | 5 | 8 | 6 | 40 | 450 |
| 44 | 30 | 5 | 8 | 7 | 40 | 450 |
| 45 | 31 | 5 | 8 | 8 | 40 | 450 |
| 46 | 77 | 8 | 5 | 5 | 40 | 141 |
| 47 | 78 | 8 | 5 | 6 | 40 | 450 |
| 48 | 79 | 8 | 5 | 7 | 40 | 450 |
| 49 | 80 | 8 | 5 | 8 | 40 | 450 |
| 50 | 42 | 6 | 7 | 5 | 42 | 61 |
| 51 | 43 | 6 | 7 | 6 | 42 | 450 |
| 52 | 44 | 6 | 7 | 7 | 42 | 450 |
| 53 | 59 | 7 | 6 | 5 | 42 | 59 |
| 54 | 60 | 7 | 6 | 6 | 42 | 450 |
| 55 | 61 | 7 | 6 | 7 | 42 | 450 |
| 56 | 47 | 6 | 8 | 6 | 48 | 450 |
| 57 | 48 | 6 | 8 | 7 | 48 | 450 |
| 58 | 49 | 6 | 8 | 8 | 48 | 450 |
| 59 | 83 | 8 | 6 | 6 | 48 | 450 |
| 60 | 84 | 8 | 6 | 7 | 48 | 450 |
| 61 | 85 | 8 | 6 | 8 | 48 | 450 |
| 62 | 64 | 7 | 7 | 6 | 49 | 450 |
| 63 | 65 | 7 | 7 | 7 | 49 | 450 |
| 64 | 68 | 7 | 8 | 6 | 56 | 156 |
| 65 | 69 | 7 | 8 | 7 | 56 | 450 |
| 66 | 70 | 7 | 8 | 8 | 56 | 450 |
| 67 | 88 | 8 | 7 | 6 | 56 | 136 |
| 68 | 89 | 8 | 7 | 7 | 56 | 450 |
| 69 | 90 | 8 | 7 | 8 | 56 | 450 |
| 70 | 94 | 8 | 8 | 7 | 64 | 450 |
| 71 | 95 | 8 | 8 | 8 | 64 | 450 |

## Appendix 3 – Parallel Versions for Search & Match Task Difficulty Levels

|  | **Search & Match Task**  **Parallel Version A** | | | | **Search & Match Task**  **Parallel Version B** | | | | |
| --- | --- | --- | --- | --- | --- | --- | --- | --- | --- |
| **No.** | **Height** | **Width** | **Tiles** | **Trials** | **Height** | **Width** | **Tiles** | **Trials** | **Version** |
|  |  |  |  |  |  |  |  |  |  |
| 01 | 4 | 4 | 4 | 450 | 4 | 4 | 4 | 450 | **Short Version (12 Levels)** |
| 02 | 5 | 4 | 4 | 450 | 4 | 5 | 4 | 450 |  |
| 03 | 4 | 5 | 5 | 450 | 5 | 4 | 5 | 450 |  |
| 04 | 5 | 5 | 4 | 103 | 5 | 5 | 4 | 103 |  |
| 05 | 5 | 5 | 5 | 450 | 5 | 5 | 5 | 450 |  |
| 06 | 6 | 4 | 4 | 339 | 4 | 6 | 4 | 432 |  |
| 07 | 4 | 6 | 5 | 450 | 6 | 4 | 5 | 450 |  |
| 08 | 6 | 4 | 6 | 450 | 4 | 6 | 6 | 450 |  |
| 09 | 6 | 5 | 5 | 450 | 5 | 6 | 5 | 450 |  |
| 10 | 5 | 6 | 6 | 450 | 6 | 5 | 6 | 450 |  |
| 11 | 6 | 6 | 5 | 442 | 6 | 6 | 5 | 442 |  |
| 12 | 6 | 6 | 6 | 450 | 6 | 6 | 6 | 450 |  |
| 13 | 7 | 4 | 4 | 47 | 4 | 7 | 4 | 51 | **Medium Version (24 Levels)** |
| 14 | 4 | 7 | 5 | 450 | 7 | 4 | 5 | 450 |  |
| 15 | 7 | 4 | 6 | 450 | 4 | 7 | 6 | 450 |  |
| 16 | 4 | 7 | 7 | 450 | 7 | 4 | 7 | 450 |  |
| 17 | 5 | 7 | 5 | 450 | 7 | 5 | 5 | 450 |  |
| 18 | 7 | 5 | 6 | 450 | 5 | 7 | 6 | 450 |  |
| 19 | 5 | 7 | 7 | 450 | 7 | 5 | 7 | 450 |  |
| 20 | 6 | 7 | 5 | 61 | 7 | 6 | 5 | 59 |  |
| 21 | 7 | 6 | 6 | 450 | 6 | 7 | 6 | 450 |  |
| 22 | 6 | 7 | 7 | 450 | 7 | 6 | 7 | 450 |  |
| 23 | 7 | 7 | 6 | 450 | 7 | 7 | 6 | 450 |  |
| 24 | 7 | 7 | 7 | 450 | 7 | 7 | 7 | 450 |  |
| 25 | 4 | 8 | 5 | 450 | 8 | 4 | 5 | 450 | **Long Version (40 Levels)** |
| 26 | 8 | 4 | 6 | 450 | 4 | 8 | 6 | 450 |  |
| 27 | 4 | 8 | 7 | 450 | 8 | 4 | 7 | 450 |  |
| 28 | 8 | 4 | 8 | 450 | 4 | 8 | 8 | 450 |  |
| 29 | 5 | 8 | 5 | 188 | 8 | 5 | 5 | 141 |  |
| 30 | 8 | 5 | 6 | 450 | 5 | 8 | 6 | 450 |  |
| 31 | 5 | 8 | 7 | 450 | 8 | 5 | 7 | 450 |  |
| 32 | 8 | 5 | 8 | 450 | 5 | 8 | 8 | 450 |  |
| 33 | 8 | 6 | 6 | 450 | 6 | 8 | 6 | 450 |  |
| 34 | 6 | 8 | 7 | 450 | 8 | 6 | 7 | 450 |  |
| 35 | 8 | 6 | 8 | 450 | 6 | 8 | 8 | 450 |  |
| 36 | 8 | 7 | 6 | 136 | 7 | 8 | 6 | 156 |  |
| 37 | 8 | 7 | 7 | 450 | 7 | 8 | 7 | 450 |  |
| 38 | 8 | 7 | 8 | 450 | 7 | 8 | 8 | 450 |  |
| 39 | 8 | 8 | 7 | 450 | 8 | 8 | 7 | 450 |  |
| 40 | 8 | 8 | 8 | 450 | 8 | 8 | 8 | 450 |  |
