# Supplementary material for: Search and Match Task: Development of a Taskified Match-3 Puzzle Game to Assess and Practice Visual Search
Source: JMIR Serious Games. 2019 May 9;7(2):e13620. doi: 10.2196/13620 (PMC6532342; doi:10.2196/13620)
Supplement: Multimedia Appendix 3 [file games_v7i2e13620_app3.docx]

# Search & Match Task – Instructions for Mac OS

## Task Distribution

**Licence:** GPL-3.0

**Version:** 1.0
**Publisher:** Alvin Chesham

## Code repository

**Name:** GitHub
**Identifier:** https://github.com/AlvinChes/SearchAndMatchTask_Mac

**Date published:** 28/01/19

## Task Description

The Search & Match Task (SMT) is a computerized visual search task based on the tile-matching match-three video game genre. The task is played on a grid-based puzzle board, filled with different types of colored shapes (tiles). The goal of the task is to find a target pattern of tiles (‘search’) on the puzzle board where two adjacent tiles can be swapped to create a vertical or horizontal line of three identical tiles (‘match’).

The SMT is designed as a single-target visual search task with multiple search target categories. The task permits control over task difficulty and collection of performance data. The SMT provides a total of 71 difficulty levels with large numbers of pre-generated trials each. Difficulty levels are defined by set size (width × height) of the puzzle board and the number of different types of colored shapes (tiles). To complete a trial for any given difficulty level, four consecutive puzzle boards with a single target must be matched. On each puzzle board, the goal is to find the target pattern on the puzzle board (‘search’) and swap two adjacent tiles to create a vertical or horizontal line of three identical tiles (‘match’).

The SMT represents an experimentally controlled puzzle game based visual search task that is thought to measure visual search and pattern recognition ability. The SMT was written using the PsychoPy stimulus presentation software to present and play the SMT and record and output performance data.

The SMT is a readily available potential tool to assess and practice visual search ability in an enjoyable and adaptive way.

Figure 1. Gameplay of the Search & Match Task (Difficulty level = (width, height, tiles) = (4, 4, 4)). For each difficulty level, trials playable trials were pre-generated as text files. A trial consists of four consecutive puzzle boards with one single target pattern (“Search”). After swapping each target pattern (“Swap”), the tiles are removed (“Remove”) and the empty cells are replaced with new tiles (“Refill”). Trials were generated such that after each refill there was only one single target pattern. The Search & Match Task is self-paced such that a new puzzle board is presented only after a valid swap that results in a match has been made.

# Step 1 – Installation PsychoPy

## Instructions for Installing PsychoPy

In order to run the Search & Match Task, the PsychoPy [1,2] experiment development program needs to be installed.

To play the Search & Match Task, please go the download link [**1.85.6 PsychoPy release**](https://github.com/psychopy/psychopy/releases/tag/1.85.6) and install the **
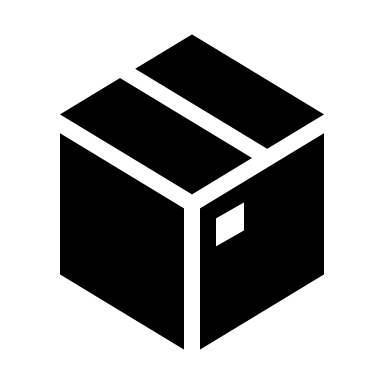
StandalonePsychoPy-1.85.6-OSX_64bit.dmg** file.

# Step 2 – Run Search & Match Task

## Opening and running the Search & Match Task

Download the SearchAndMatchTask_Mac-master file from

[**https://github.com/AlvinChes/SearchAndMatchTask_Mac/archive/master.zip**](https://github.com/AlvinChes/SearchAndMatchTask_Mac/archive/master.zip)

and extract to a location of your choice.

Start PsychoPy and open the Psychopy Coder view shell (View > Open Coder view).

From within the Search&MatchTask folder, open the **SearchMatchTask_main.py** file in the Coder view shell (Use File > Open or Cmd+O) and launch the Search & Match Task by clicking the “run” button (Tools > Run or Cmd+R).


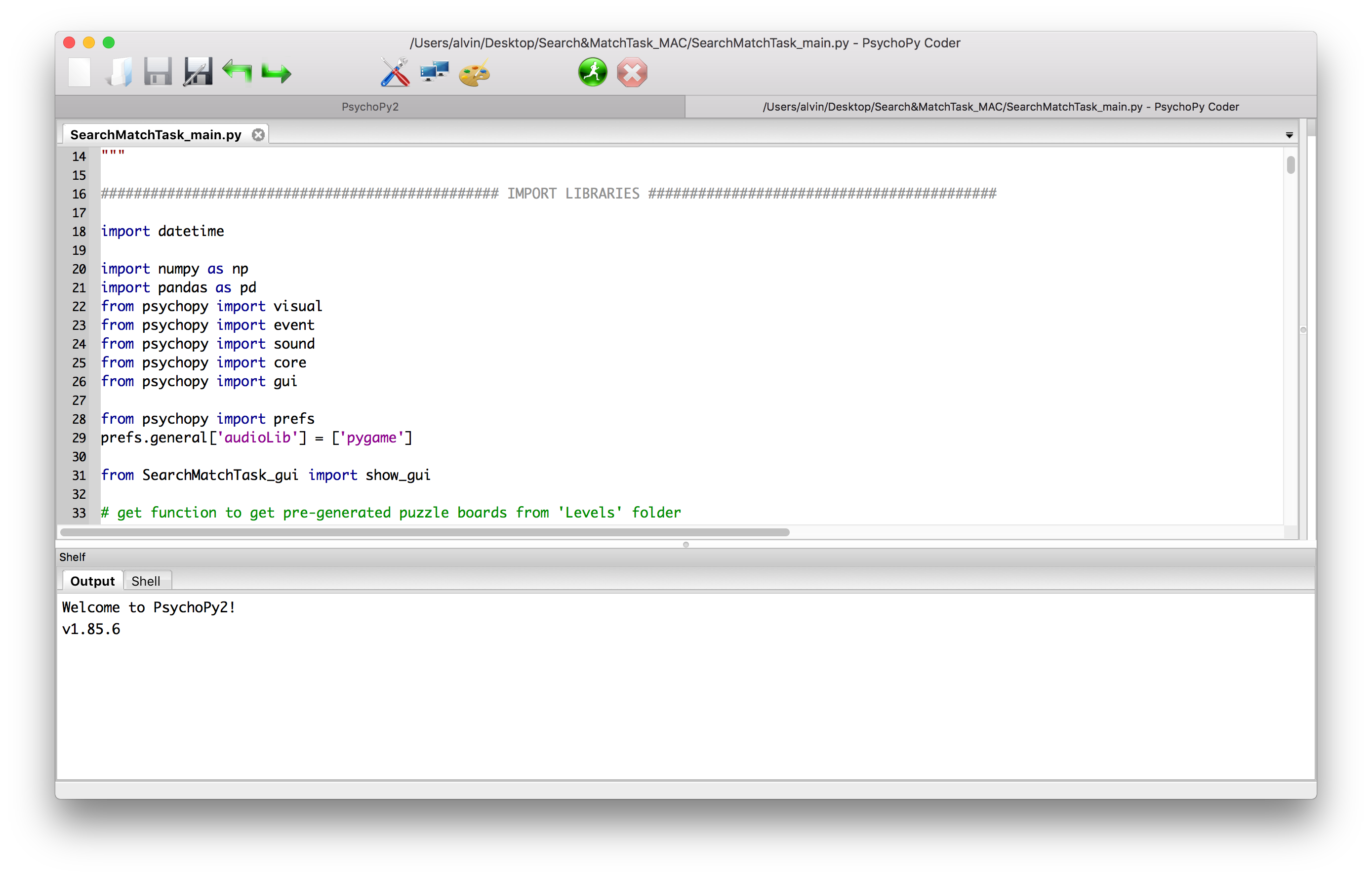


### Troubleshoot the event.py file

Upon running the file for the first time an error will occur and the task will crash:


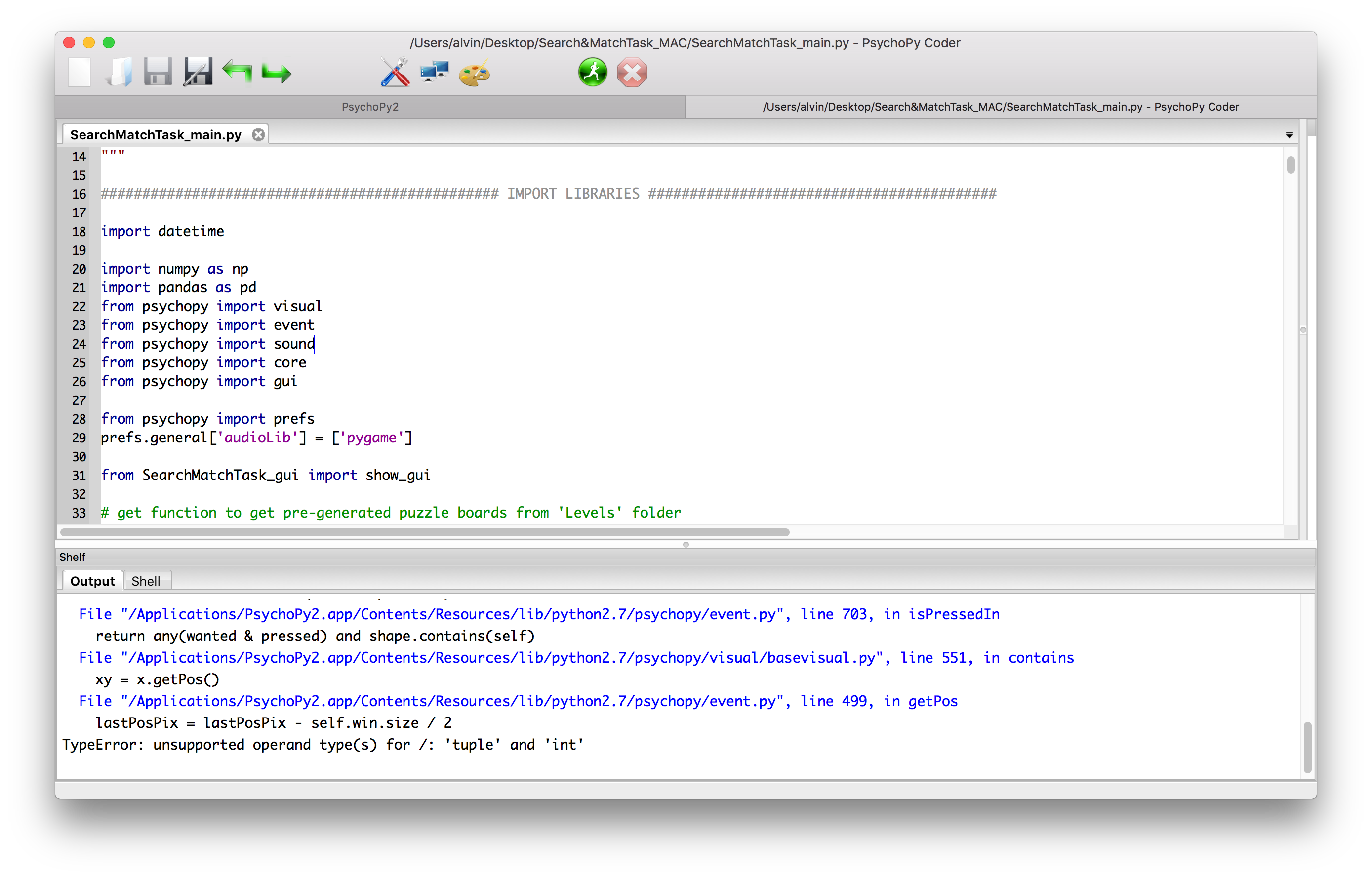


Navigate to the line/file where the error occurred by clicking on the traceback:

**File "/Applications/PsychoPy2.app/Contents/Resources/lib/python2.7/psychopy/event.py", line 499, in getPos**

This will open the event.py file at code line 499. The error concerns the line:

lastPosPix = lastPosPix - self.win.size / 2


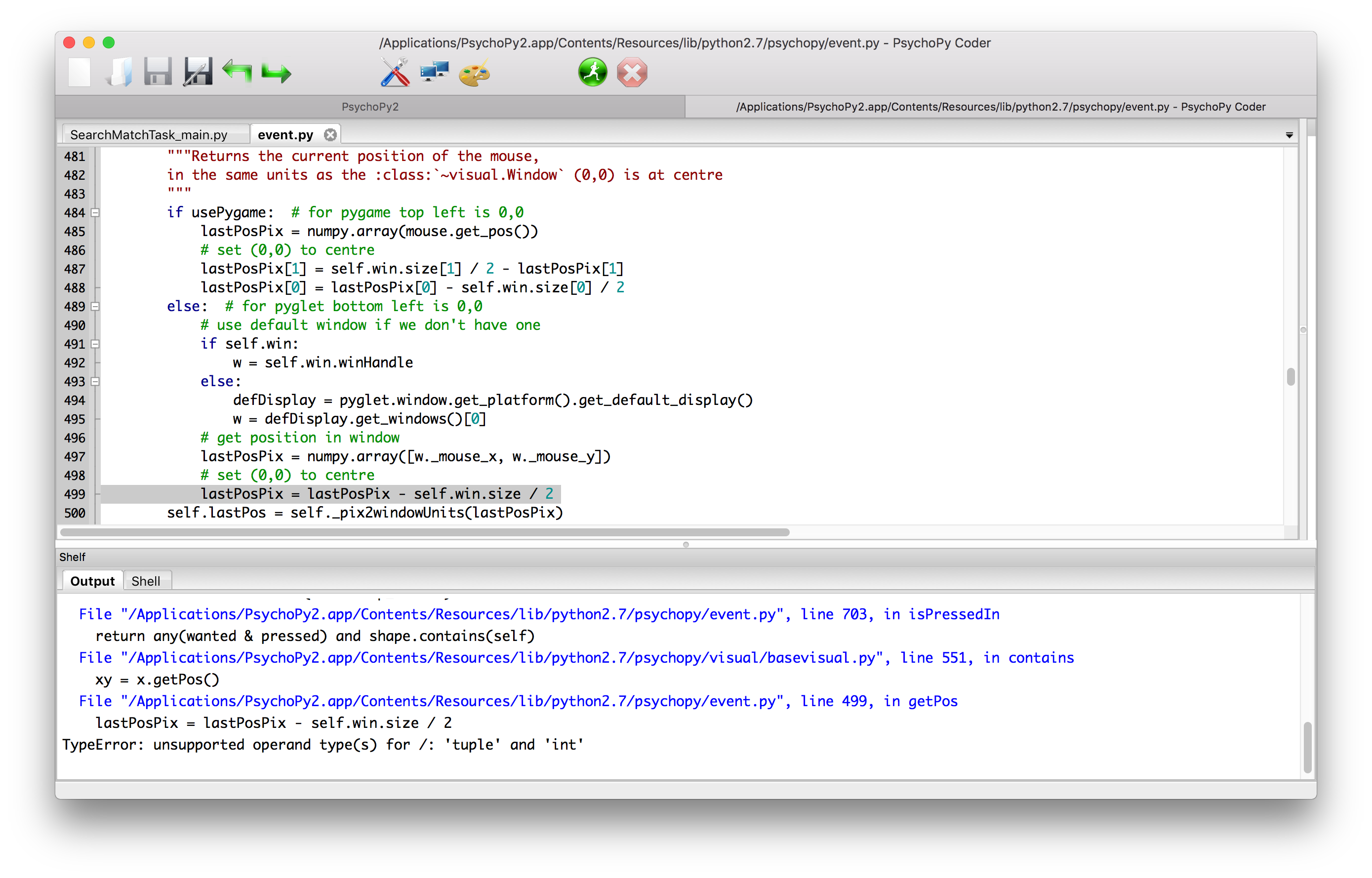


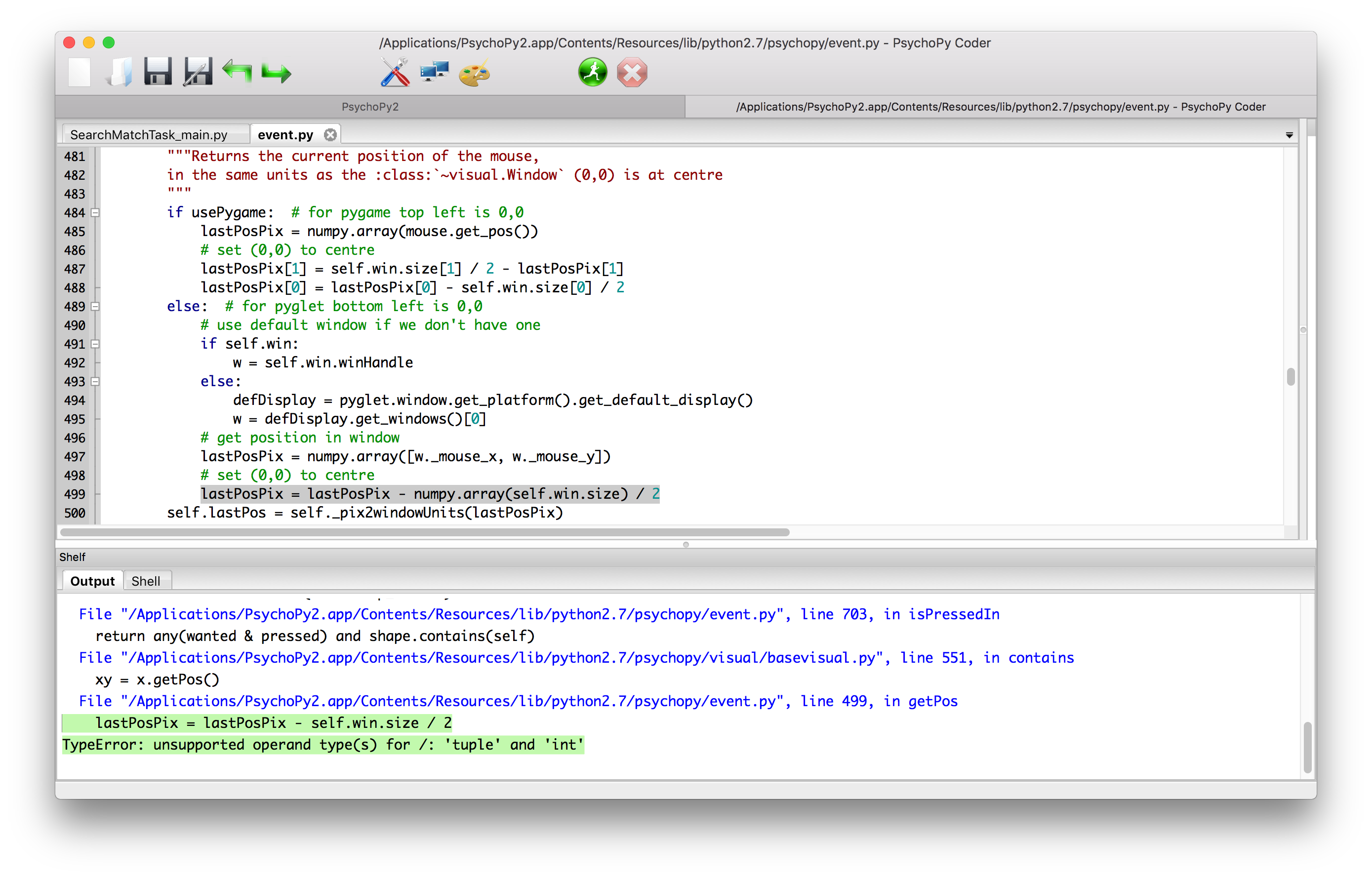


Replace the line lastPosPix = lastPosPix - self.win.size / 2 with

lastPosPix = lastPosPix - numpy.array(self.win.size) / 2 and run the event.py file in order to save the change.

Upon completing this change, the Search & Match Task can be run without any issues.

# Step 3 - Task settings for the Search & Match Task

Running the **SearchMatchTask_main.py** file brings up the configuration interface of the Search & Match Task. The interface allows experimenters to input standard Participant Information (i.e. ID, age and gender). Settings for the Search & Match Task can be configured (see Figure 1).


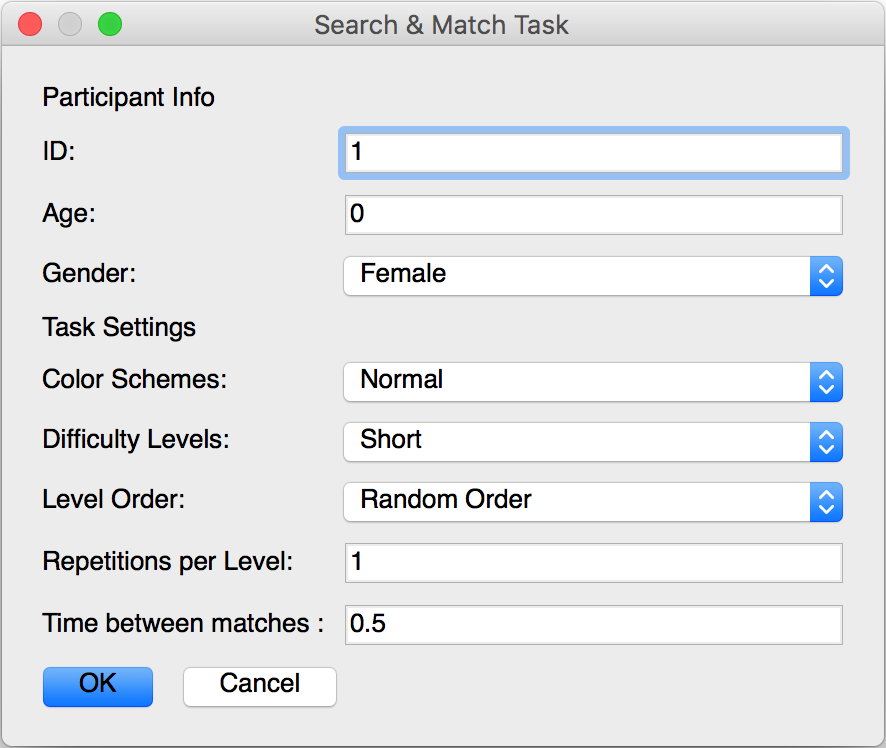


Figure 1. Search & Match Task Settings

- The “*Color schemes*” setting concerns the color palette for the colored shapes on the puzzle board. There are two available color palettes: the “Discriminable Color Palette” includes eight highly discriminable colors that were previously used in a visual search task [3]. Also, a “Color-blind friendly Palette” optimized for color-blind individuals [4] is available (see Figure 2).

Figure 2. Color Schemes for the colored shapes (tiles) in the Search & Match Task. Available color schemes include the “Discriminable Color Palette” (top row) and the “Color-blind friendly ”

- In the “*Difficulty Levels*” setting, the sets of difficulty levels for the Search & Match Task can be selected. there are four folders of difficulty levels to choose from: a “Short”, “Medium”, “Long” and “Custom” folder. The short version includes difficulty levels 4_4_4 to 6_6_6 (12 levels) , the medium version includes levels 4_4_4 to 7_7_7 (24 levels) and the long version includes levels 4_4_4 to 8_8_8 (40 levels) (see Appendix 1). The “Custom” folder setting allows researchers to create a custom set of difficulty levels where selected difficulty levels can be added by copy paste.
- In the “*Level Order*” setting, the order the difficulty levels are presented, can be specified. “Random order” will present the different difficulty levels in a randomized fashion, whereas “Order in file” will present the levels sequentially by the sequential order in the respective difficulty level folder.
- The “*Repetitions per Level*” setting allows to specify the number of trials presented for each difficulty level selected in the “Difficulty Levels” setting. For example, a value of 1 will randomly pick 1 trial with four consecutive matches for every difficulty level.
- The “*Match Presentation Time*” allows to set the time period (in seconds) a match (i.e. a line of three identical tiles) is shown after tiles of a target pattern have been correctly swapped.

# Step 4 - Search & Match Task Instruction

## General Introduction

*Welcome to the Search & Match Task. This task examines your visual search ability.*

*In this task you are presented puzzle boards filled with different colored shapes called ‘tiles’. Your goal is to create vertical or horizontal lines of three identical tiles called a ”match”.*

*You can make a match by swapping the position of two adjacent tiles by clicking on both of them using the mouse. Tiles can be swapped in all four cardinal directions but not diagonally.*

*When the swapped tiles do not create a match, the will bounce back to their initial place. When the swapped tiles create a match, the matched tiles are removed, and new tiles drop in their place.*

## Practice Trial Instruction

*To help you understand the visual search task, you can play three practice trials.*

*On each practice trial, your task is to find a pattern of three identical tiles where you can swap two adjacent tiles to create a line of three identical tiles. Each trial ends when you have made four consecutive matches.*

*Basically, there are three types of patterns that you need to look out for to make a match (see Figure 3). Note that matches can occur at any location on the puzzle board using any of the different colored tiles.*

Figure 3. Basic target patterns in the Search & Match Task based on [5–7]. There are three basic configurations of tiles that can be aligned to a line of three identical tiles by swapping two adjacent tiles: the “i-Pattern” where a tile is moved towards a pair of two adjacent identical tiles, the “J-Pattern” where a tile is moved in line with two adjacent identical tiles and the “V-Pattern” where a tile is moved in line between two identical tiles. These basic target patterns can occur in all possible orientations and mirrored versions.

*To help you understand the Search & Match Task, you can play three practice trials.*

*On each practice trial, your task is to find a pattern of three identical tiles where you can swap two adjacent tiles to create a line of three identical tiles. Each trial ends when you have made four consecutive matches.*

## Test Trial Instruction

*Congratulations, you've completed the practice trials. You can now play a number of levels of the Search & Match Task. Try to be as quick and accurate as possible. Do not hesitate to use the hint button when needed.*

*Press the enter key to start the practice trials.*

## Playing the Search & Match Task

In the Search & Match Task, a grid-based puzzle board with a given set size (width × height) randomly filled with tiles from a given number of unique tile types (tiles) are shown on a grey background. To solve a trial of any given level, 4 consecutive valid moves (‘matches’) must be made. After completing four valid moves, an inter-trial window with a “Continue” button is shown. After clicking “Continue” the next trial starts (see Figure 4).


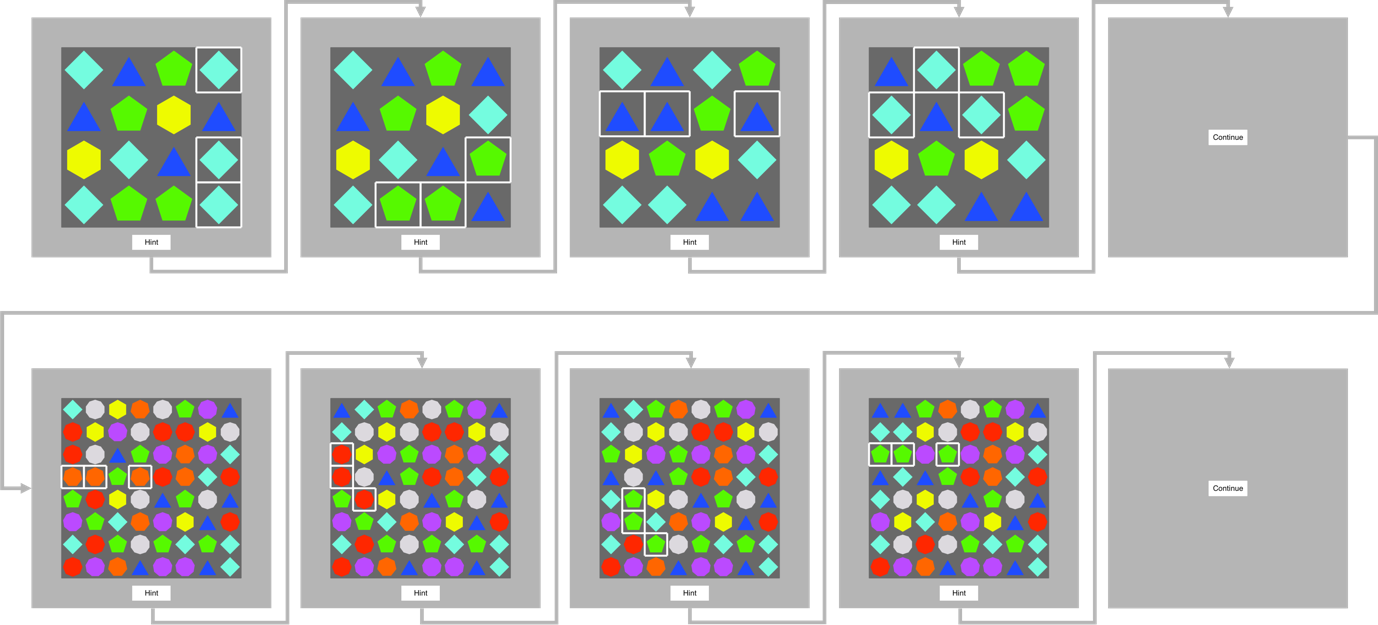


Figure 4. Trial sequence of the Search & Match Task. Each trial of a given level starts with a start board with a given width, height and number of tile types. To complete a trial, four consecutive target patterns (highlighted with white border) must be searched and matched. The top row depicts the lowest level (width = 4, height = 4, types of tiles = 4), the bottom row shows highest level (width = 8, height = 8, types of tiles = 8).

Moves can be made by exchanging the position of two adjacent tiles (‘swap’) in any of the four cardinal directions by clicking on them with the mouse. A move is valid when it creates a vertical or horizontal alignment of three identical tiles (‘match’) by swapping two adjacent tiles (see Figure 3).

Figure 5. Valid move. In a valid move a tile that is part of a target pattern (highlighted with white border, see Figure 7 for reference) is switched with an adjacent tile to correctly make an alignment of three identical tiles (‘match’). The match is then removed and a new target pattern must be found and matched.

Invalid moves, are moves that do not create a ‘match’, are not allowed and in this case the swapped tiles will bounce back to their initial place.

Figure 6. Invalid move. In invalid moves a tile that does not result in a vertical or horizontal alignment of three identical tiles is switched with an adjacent tile (see Distractor Patterns in Figure 7 for reference). After an invalid move was made, the wrongly switched tiles bounce back into their original position and the puzzle board is shown until the target pattern (highlighted with white border) is matched.

# Step 5 - Search & Match Task Output Data

## Performance Data Collection

Performance data is recorded as a csv file saved inside the **SearchAndMatchTask_Mac/DATA** folder. In the Search & Match Task the following outcomes and variables are recorded (see Table 1 for reference):

| *Condition* | Indicates whether the played trial is a practice or test trial  (‘Practice Trial’ or ‘Test trial’) |
| --- | --- |
| *Version* | Indicates the difficulty level version (see Table 2). |
| *Level Trial* | Order in which the difficulty levels were presented. |
| *Width* | Number of horizontal cells in the puzzle board grid. |
| *Height* | Number of vertical cells in the puzzle board grid. |
| *Tiles* | Number of different types of colored shapes on the puzzle board. |
| *Level File Number* | Refers to the randomly selected trial selected from the pre-generated trials within the respective difficulty level folder. |
| *Move Trial* | Order of the played moves for each difficulty level. Includes both valid and false moves. |
| *Move*  *Accuracy* | Outcome of interaction with a trial. Includes valid moves, i.e. swaps between tiles that result in a match and false moves, i.e. swaps between adjacent or non-adjacent tiles that do not result in a match (‘valid’, 'invalid-adjacent', 'invalid-non-adjacent'). |
| *Hint* | Indicates whether a hint was used to find the target pattern or not. |
| *Search*  *Time* | Response time (seconds) from onset of a puzzle board to completion of a move. Response times are calculated for valid and false moves. |
| *Number of Valid Moves* | Total number of valid moves per difficulty level. |
| *Number of Invalid Moves* | Total number of invalid moves per difficulty level. |
| *Number of Hints* | Total number of hints used per difficulty level. |
| *Target Pattern Type* | Indicates the visual search target type made up by a spatial relation among three identical tiles that can be swapped to make a match.  There are 16 types of target pattern categories based on three basic types of patterns (see Figure 2). |
| *Target pattern coordinates* | Coordinates of the tiles that constitute a target pattern (see Figure 2) in the puzzle array (width x height). A list of four tuples is provided. The last tuple in this list represents the tile that must be swapped (see red tiles in Figure 2), whereas the others include the coordinates of the three identical tiles (see green tiles in Figure 2). |
| *Clicked Tiles Coordinates* | Coordinates of the two tiles that were clicked to make a swap. Includes clicked tiles for both valid and false moves. |

| Difficulty Level-specific Variables | | | | | | | Move-specific Variables | | | | | | | | | |
| --- | --- | --- | --- | --- | --- | --- | --- | --- | --- | --- | --- | --- | --- | --- | --- | --- |
| Condition | Version | Level Trial | Width | Height | Tiles | Level File Number | Move Trial | Move Accuracy | Hint | Search Time | Number Valid Moves | Number False Moves | Number Hints | Target Pattern | Target Pattern Coordinates | Clicked Tiles Coordinates |
| Practice Trial | Levels_Practice | 1 | 4 | 4 | 4 | 411 | 1 | valid | no hint | 4.803220034 | 1 | 0 | 0 | vertical_i_top | [(2, 0), (3, 0), (0, 0), (1, 0)] | [(0, 0), (0, 1)] |
| Practice Trial | Levels_Practice | 1 | 4 | 4 | 4 | 411 | 2 | valid | no hint | 5.252591848 | 2 | 0 | 0 | horizontal_j_bottom_right | [(2, 0), (2, 1), (3, 2), (2, 2)] | [(2, 3), (2, 2)] |
| Practice Trial | Levels_Practice | 1 | 4 | 4 | 4 | 411 | 3 | valid | no hint | 2.685039043 | 3 | 0 | 0 | vertical_i_top | [(2, 2), (3, 2), (0, 2), (1, 2)] | [(2, 0), (2, 1)] |
| Practice Trial | Levels_Practice | 1 | 4 | 4 | 4 | 411 | 4 | valid | no hint | 2.852303028 | 4 | 0 | 0 | horizontal_i_left | [(0, 2), (0, 3), (0, 0), (0, 1)] | [(1, 0), (0, 0)] |
| Test Trial | Levels_Long_A | 1 | 7 | 7 | 7 | 429 | 1 | valid | no hint | 13.258183 | 1 | 0 | 0 | vertical_v_right | [(1, 5), (3, 5), (2, 6), (2, 5)] | [(5, 2), (6, 2)] |
| Test Trial | Levels_Long_A | 1 | 7 | 7 | 7 | 429 | 2 | valid | no hint | 2.751899004 | 2 | 0 | 0 | horizontal_v_top | [(1, 3), (1, 5), (0, 4), (1, 4)] | [(4, 1), (4, 0)] |
| Test Trial | Levels_Long_A | 1 | 7 | 7 | 7 | 429 | 3 | valid | no hint | 2.634831905 | 3 | 0 | 0 | vertical_v_right | [(0, 5), (2, 5), (1, 6), (1, 5)] | [(5, 1), (6, 1)] |
| Test Trial | Levels_Long_A | 1 | 7 | 7 | 7 | 429 | 4 | valid | no hint | 2.735810995 | 4 | 0 | 0 | vertical_j_top_left | [(1, 6), (2, 6), (0, 5), (0, 6)] | [(5, 0), (6, 0)] |
| Test Trial | Levels_Long_A | 2 | 5 | 6 | 5 | 202 | 1 | valid | hint | 7.137173891 | 1 | 0 | 1 | vertical_i_bottom | [(0, 4), (1, 4), (3, 4), (2, 4)] | [(4, 2), (4, 3)] |
| Test Trial | Levels_Long_A | 2 | 5 | 6 | 5 | 202 | 2 | valid | no hint | 2.518749952 | 2 | 0 | 1 | horizontal_j_top_left | [(1, 3), (1, 4), (0, 2), (1, 2)] | [(2, 1), (2, 0)] |
| Test Trial | Levels_Long_A | 2 | 5 | 6 | 5 | 202 | 3 | valid | no hint | 3.653833866 | 3 | 0 | 1 | vertical_v_left | [(1, 4), (3, 4), (2, 3), (2, 4)] | [(3, 2), (4, 2)] |
| Test Trial | Levels_Long_A | 2 | 5 | 6 | 5 | 202 | 4 | valid | no hint | 3.619835138 | 4 | 0 | 1 | horizontal_j_top_left | [(2, 3), (2, 4), (1, 2), (2, 2)] | [(2, 1), (2, 2)] |
| Test Trial | Levels_Long_A | 3 | 7 | 5 | 7 | 236 | 1 | invalid-adjacent | no hint | 3.869632006 | 1 | 1 | 0 | vertical_i_top | [(3, 4), (4, 4), (1, 4), (2, 4)] | [(3, 1), (3, 2)] |
| Test Trial | Levels_Long_A | 3 | 7 | 5 | 7 | 236 | 2 | valid | hint | 26.53005195 | 1 | 1 | 1 | vertical_i_top | [(3, 4), (4, 4), (1, 4), (2, 4)] | [(4, 1), (4, 2)] |
| Test Trial | Levels_Long_A | 3 | 7 | 5 | 7 | 236 | 3 | valid | no hint | 2.517868042 | 2 | 1 | 1 | horizontal_j_bottom_right | [(0, 3), (0, 4), (1, 5), (0, 5)] | [(5, 1), (5, 0)] |
| Test Trial | Levels_Long_A | 3 | 7 | 5 | 7 | 236 | 4 | valid | no hint | 2.034838915 | 3 | 1 | 1 | vertical_i_bottom | [(0, 4), (1, 4), (3, 4), (2, 4)] | [(4, 3), (4, 2)] |
| Test Trial | Levels_Long_A | 3 | 7 | 5 | 7 | 236 | 5 | valid | no hint | 3.586359978 | 4 | 1 | 1 | vertical_j_bottom_right | [(2, 4), (3, 4), (4, 5), (4, 4)] | [(5, 4), (4, 4)] |
| Test Trial | Levels_Long_A | 4 | 5 | 7 | 6 | 417 | 1 | valid | no hint | 3.4535079 | 1 | 0 | 0 | vertical_v_left | [(0, 3), (2, 3), (1, 2), (1, 3)] | [(2, 1), (3, 1)] |
| Test Trial | Levels_Long_A | 4 | 5 | 7 | 6 | 417 | 2 | invalid-adjacent | no hint | 3.88628602 | 2 | 1 | 0 | horizontal_i_left | [(2, 3), (2, 4), (2, 1), (2, 2)] | [(2, 2), (3, 2)] |
| Test Trial | Levels_Long_A | 4 | 5 | 7 | 6 | 417 | 3 | valid | no hint | 6.169538975 | 2 | 1 | 0 | horizontal_i_left | [(2, 3), (2, 4), (2, 1), (2, 2)] | [(2, 2), (1, 2)] |
| Test Trial | Levels_Long_A | 4 | 5 | 7 | 6 | 417 | 4 | valid | no hint | 3.269062996 | 3 | 1 | 0 | horizontal_i_right | [(2, 0), (2, 1), (2, 3), (2, 2)] | [(3, 2), (2, 2)] |
| Test Trial | Levels_Long_A | 4 | 5 | 7 | 6 | 417 | 5 | valid | no hint | 2.535349846 | 4 | 1 | 0 | horizontal_j_top_left | [(3, 1), (3, 2), (2, 0), (3, 0)] | [(0, 2), (0, 3)] |

Table 1. Raw output file for the Search & Match Task. Data is recorded for each move of every difficulty level. Data entries include the task condition (practice or test trial), the puzzle difficulty version (see Appendix 1). Difficulty level specific data includes the level trial number, height and width of the puzzle board and the number of tile types. For each level the number of the randomly selected pre-generated trial in the file is provided. On the move level, the move trial number, accuracy of the move (valid or false move), whether a hint was used target search time is recorded. The number of valid and false moves, and used hints is summed up for each difficulty level. Finally, the type of target pattern (see Figure 2), the coordinates of the tiles of the target pattern and the coordinates of the clicked tiles are provided.

Figure 4. Target pattern **categories** based on [5–7].: The green tile can be swapped with the respective opposite red tile to make a line of three red tiles (‘match’). There are three basic target patterns that can be matched by moving a tile diagonal from a pair of identical pieces (“J”-patterns), between two identical tiles (“V”-patterns) and towards a pair of tiles (“i”-patterns). There are 16 different possible types of target patterns.

Table 2. Parallel Versions for Search & Match Difficulty Levels

|  | **Search & Match Task**  **Parallel Version A** | | | |  | **Search & Match Task**  **Parallel Version B** | | | |  |
| --- | --- | --- | --- | --- | --- | --- | --- | --- | --- | --- |
| **No.** | **Height** | **Width** | **Tiles** | **Trials** |  | **Height** | **Width** | **Tiles** | **Trials** | **Version** |
| 01 | 4 | 4 | 4 | 450 |  | 4 | 4 | 4 | 450 | **Short Version (12 Levels)** |
| 02 | 5 | 4 | 4 | 450 |  | 4 | 5 | 4 | 450 |  |
| 03 | 4 | 5 | 5 | 450 |  | 5 | 4 | 5 | 450 |  |
| 04 | 5 | 5 | 4 | 103 |  | 5 | 5 | 4 | 103 |  |
| 05 | 5 | 5 | 5 | 450 |  | 5 | 5 | 5 | 450 |  |
| 06 | 6 | 4 | 4 | 339 |  | 4 | 6 | 4 | 432 |  |
| 07 | 4 | 6 | 5 | 450 |  | 6 | 4 | 5 | 450 |  |
| 08 | 6 | 4 | 6 | 450 |  | 4 | 6 | 6 | 450 |  |
| 09 | 6 | 5 | 5 | 450 |  | 5 | 6 | 5 | 450 |  |
| 10 | 5 | 6 | 6 | 450 |  | 6 | 5 | 6 | 450 |  |
| 11 | 6 | 6 | 5 | 442 |  | 6 | 6 | 5 | 442 |  |
| 12 | 6 | 6 | 6 | 450 |  | 6 | 6 | 6 | 450 |  |
| 13 | 7 | 4 | 4 | 47 |  | 4 | 7 | 4 | 51 | **Medium Version (24 Levels)** |
| 14 | 4 | 7 | 5 | 450 |  | 7 | 4 | 5 | 450 |  |
| 15 | 7 | 4 | 6 | 450 |  | 4 | 7 | 6 | 450 |  |
| 16 | 4 | 7 | 7 | 450 |  | 7 | 4 | 7 | 450 |  |
| 17 | 5 | 7 | 5 | 450 |  | 7 | 5 | 5 | 450 |  |
| 18 | 7 | 5 | 6 | 450 |  | 5 | 7 | 6 | 450 |  |
| 19 | 5 | 7 | 7 | 450 |  | 7 | 5 | 7 | 450 |  |
| 20 | 6 | 7 | 5 | 61 |  | 7 | 6 | 5 | 59 |  |
| 21 | 7 | 6 | 6 | 450 |  | 6 | 7 | 6 | 450 |  |
| 22 | 6 | 7 | 7 | 450 |  | 7 | 6 | 7 | 450 |  |
| 23 | 7 | 7 | 6 | 450 |  | 7 | 7 | 6 | 450 |  |
| 24 | 7 | 7 | 7 | 450 |  | 7 | 7 | 7 | 450 |  |
| 25 | 4 | 8 | 5 | 450 |  | 8 | 4 | 5 | 450 | **Long Version (40 Levels)** |
| 26 | 8 | 4 | 6 | 450 |  | 4 | 8 | 6 | 450 |  |
| 27 | 4 | 8 | 7 | 450 |  | 8 | 4 | 7 | 450 |  |
| 28 | 8 | 4 | 8 | 450 |  | 4 | 8 | 8 | 450 |  |
| 29 | 5 | 8 | 5 | 188 |  | 8 | 5 | 5 | 141 |  |
| 30 | 8 | 5 | 6 | 450 |  | 5 | 8 | 6 | 450 |  |
| 31 | 5 | 8 | 7 | 450 |  | 8 | 5 | 7 | 450 |  |
| 32 | 8 | 5 | 8 | 450 |  | 5 | 8 | 8 | 450 |  |
| 33 | 8 | 6 | 6 | 450 |  | 6 | 8 | 6 | 450 |  |
| 34 | 6 | 8 | 7 | 450 |  | 8 | 6 | 7 | 450 |  |
| 35 | 8 | 6 | 8 | 450 |  | 6 | 8 | 8 | 450 |  |
| 36 | 8 | 7 | 6 | 136 |  | 7 | 8 | 6 | 156 |  |
| 37 | 8 | 7 | 7 | 450 |  | 7 | 8 | 7 | 450 |  |
| 38 | 8 | 7 | 8 | 450 |  | 7 | 8 | 8 | 450 |  |
| 39 | 8 | 8 | 7 | 450 |  | 8 | 8 | 7 | 450 |  |
| 40 | 8 | 8 | 8 | 450 |  | 8 | 8 | 8 | 450 |  |
