# Supplementary material for: Search and Match Task: Development of a Taskified Match-3 Puzzle Game to Assess and Practice Visual Search
Source: JMIR Serious Games. 2019 May 9;7(2):e13620. doi: 10.2196/13620 (PMC6532342; doi:10.2196/13620)
Supplement: Multimedia Appendix 4 [file games_v7i2e13620_app4.zip › SearchAndMatchTask_Win-master/Instructions/Instructions_framing.pdf]

TASK 1

General Introduction

Welcome and thank you for participating in our experiment. The aim of the study is to examine your decision-making about gambles

You will play a game in which on every trial you are offered some money to play with. You are asked to choose between two options: a sure option which means a guaranteed amount of money or you can decide to play a lottery with specific odds. The initial amount you will get and the odds differ from trial to trial.

You are playing for real money at all times so your task is to be attending through the entire experiment which would allow you to maximize your final score. At the end of the experiment you will receive a sum proportional to how much you will win during the experiment

Instructions:

Part 1 (general info):

On each trial, you will see a screen with a '+' symbol. This symbol indicates you are about to be presented with the initial amount we offer you to play with on that particular trial.

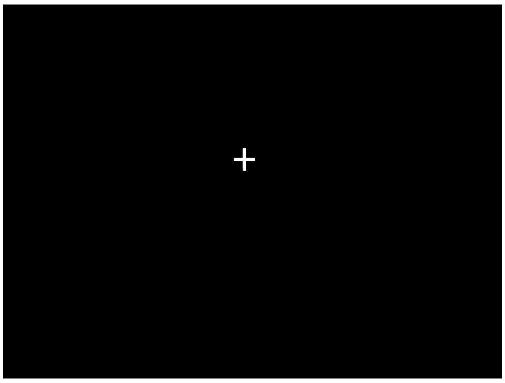

After that you will see a screen saying "Receive …" - this means you receive an initial amount of money that you will play for on this trial.

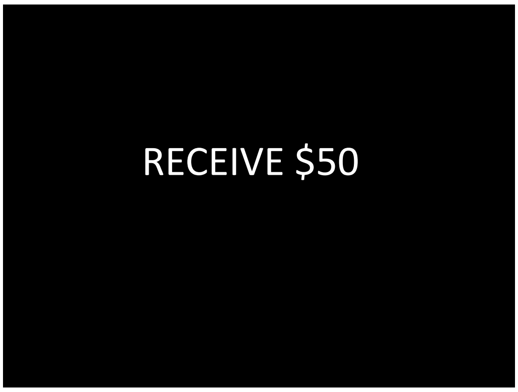

Note that you do not get to keep all this money, but you have to decide on the next screen like e.g. below between two options

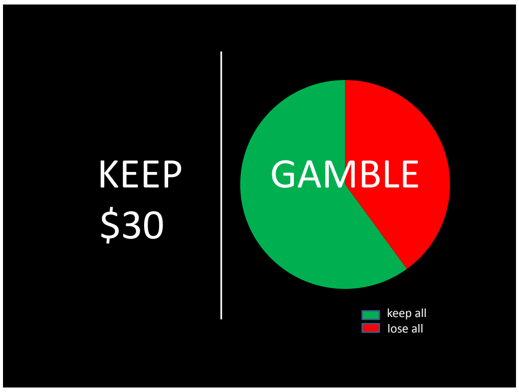

This means that you have 2 options: one option is to take a portion of the initial sum for sure: e.g. here this is $30 out of the $50 that you received initially (this is the sure option)
OR You can take a gamble which is represented by a pie chart on the right side. This means that with the gamble, you have a chance of winning the entire $50 but you also have a chance that you will get nothing.

- Your chance of winning the total amount is the green section of the pie chart and your chance of losing it is the red section.
- You are reminded what red and green means just below the pie chart

You make your selection by pressing the key

Press the key when you have made the decision, and please make the decision within 4 seconds.

Try not to miss any decisions- if this happens we will discard that trial. But if this happens more than a few times, this will reduce the amount you can win at the end.

Part 2 (types of trials)

Note that there are a few different types of trials. In the example we have described, it says you receive $50 and you get to keep $30 (or gamble for the full $50).   In other trials, however, it might say you receive $100 and then it might say you lose $40.

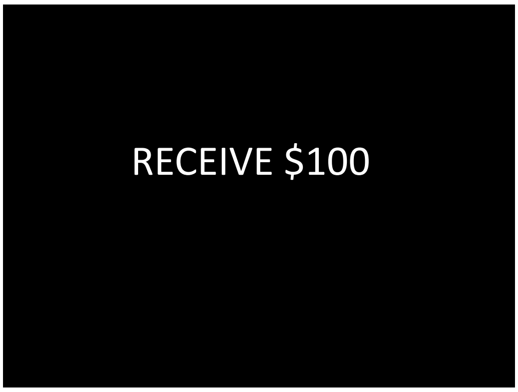

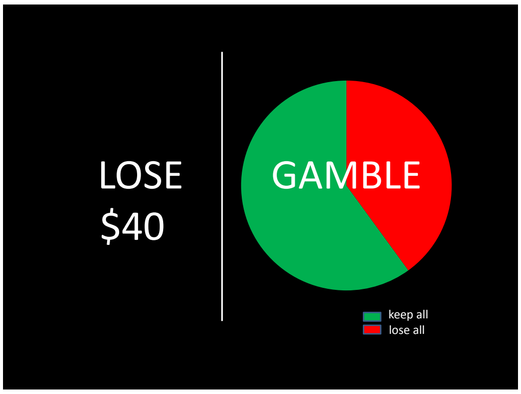

During these trials, the $40 represents the amount you lose for sure from the initial starting amount (in this case $100) - so you keep the rest of the amount which in this case would be $60 if you were to select the sure option (or you chose the gamble).

So, it is very important for you to pay attention to what the screen tells you and what options you have.

Part 3 (strategy)

There is not necessarily any perfect strategy to play this game but what you will realize soon is that it may not be a great idea to play the lottery on every trial, or to take the sure option all the time since the options change every time
