# Supplementary figures and images for: Search and Match Task: Development of a Taskified Match-3 Puzzle Game to Assess and Practice Visual Search
Source: JMIR Serious Games. 2019 May 9;7(2):e13620. doi: 10.2196/13620 (PMC6532342; doi:10.2196/13620)

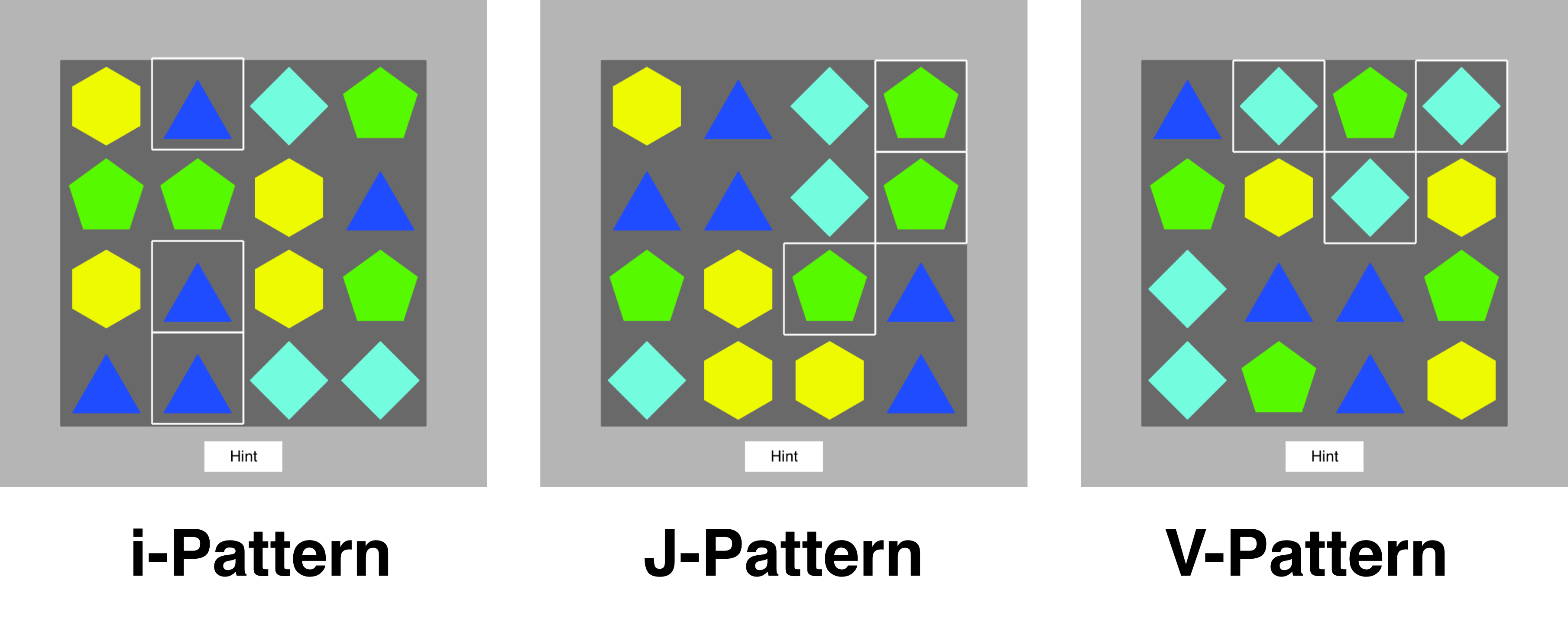

Supplement: Multimedia Appendix 4 [file games_v7i2e13620_app4.zip › SearchAndMatchTask_Win-master/Instructions/BasicTargetPatterns.png]

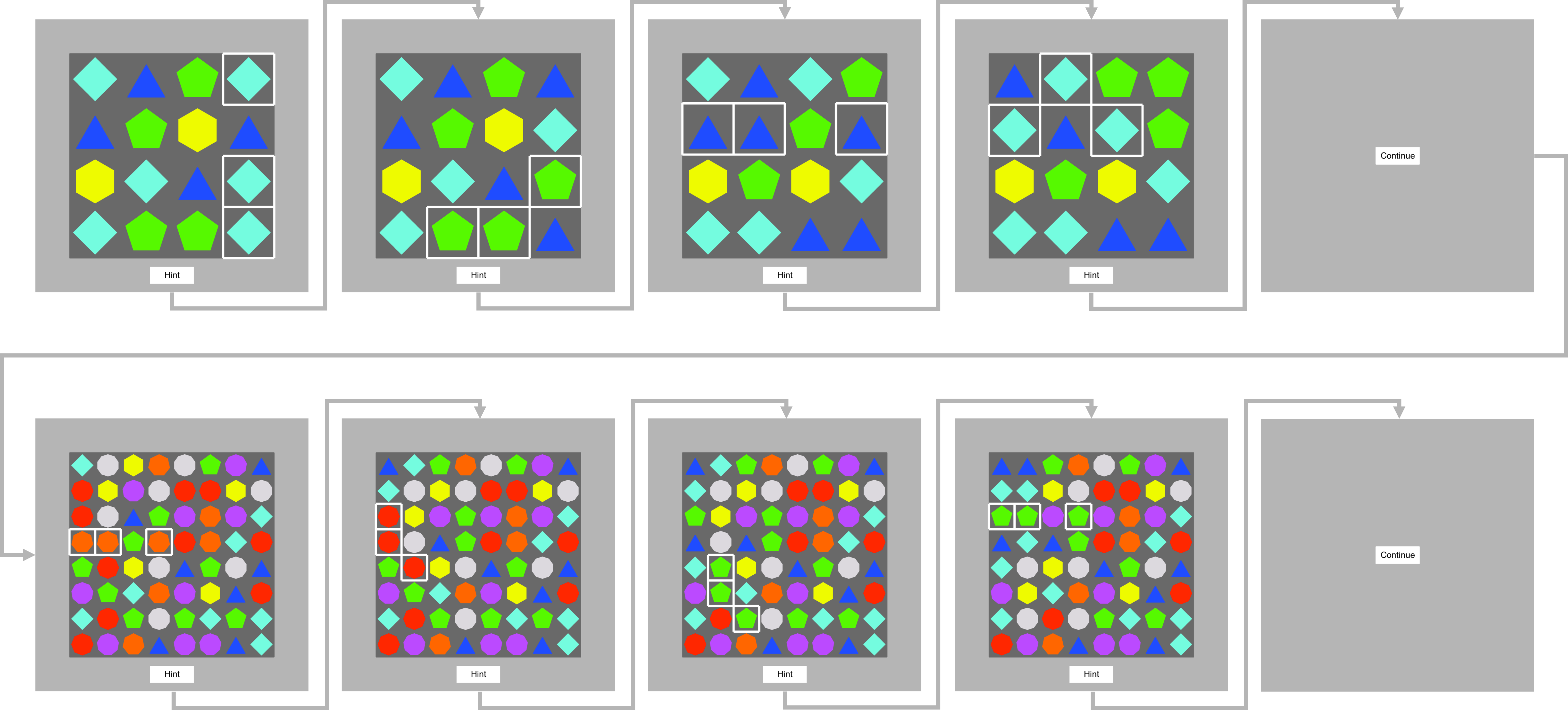

Supplement: Multimedia Appendix 5 [file games_v7i2e13620_app5.zip › SearchAndMatchTask_Mac-master/Picture1.png]
